# Illustrating Fuego: the particular challenges and richness of using arts-based participatory methods to communicate experiences of volcanic disaster

**DOI:** 10.1186/s13617-025-00149-0

**Published:** 2025-02-10

**Authors:** Ailsa K. Naismith

**Affiliations:** 1 School of Earth Sciences, Wills Memorial Building, University of Bristol, Bristol, UK; 2 Earth Observatory of Singapore, Nanyang Technological University, Singapore, Singapore

**Keywords:** Zines, Arts-based methods, Illustration, Participatory research, Disaster, Volcanic eruption

## Abstract

Although disasters disproportionately affect vulnerable people, their experiences often go untold. Disaster researchers increasingly use participatory research methods to recognize and amplify these missing voices. Arts-based participatory methods promise both empowerment for local people and opportunity for researchers to work creatively and reflexively. They also present challenges of participation and representation. This paper describes the process of co-producing a “zine” describing local people’s experiences of eruptions of Fuego volcano (Guatemala) and the impacts of these eruptions that caused disaster. The zine is narrated by local people and is illustrated by the researcher (me) who conducted semi-structured interviews of residents to better understand their experiences. The motivations for this project are explored together with lessons learned in turning participatory research design into practice. Taking a constructively critical perspective on my process, I document challenges I encountered in community participation and representation and discuss how they may be addressed in project design and execution. I also describe the tensions and value in occupying both researcher and artist roles. This paper describes an open method of research exploration where the opportunities and limitations of visual representation to contribute to shared and widening understandings of volcanic disaster experience is documented and reflected upon.

**Supplementary Information:**

The online version contains supplementary material available at 10.1186/s13617-025-00149-0.

## Introduction

Participatory research aims to “give members of marginalized groups a voice, or to enable them to make their voices heard” (Bergold and Thomas 2012, p. 202). This is particularly relevant for disasters related to natural hazards, where marginalized people are disproportionately affected but often overlooked. Disaster researchers may choose participatory methods because, rather than treating people as a homogenous group of “disaster victims”, these methods capture their diverse experiences (Sou and Hall 2021) and offer trauma-sensitive alternatives where standard approaches such as interviews might be unwelcome (Espinoza et al. 2019). Participatory methods also promise researchers greater flexibility and reflexivity than conventional research designs (Bergold and Thomas 2012; Childs et al. 2017). However, participatory methods pose their own challenges: working with communities who have experienced disasters requires time, sensitivity, and attention to ethics. Control of the research process is a complex issue (Le De et al. 2014); although community control is often identified as the highest ideal of participation (because those most directly impacted are knowledgeable of risks and best suited to guide initiatives), in practice this is rarely achieved (Nkombi & Wentik, 2022). The definition of “success” changes with stakeholder perspective (Bergold and Thomas 2012), and practical hurdles include participant time or energy constraints and misaligned project conceptions or expectations. Despite these myriad challenges, the benefits of participatory research methods endure. These benefits include greater opportunity for dialogue, more equity between researcher and participants (“respecting and understanding the people with and for whom researchers work”, Cornwall and Jewkes 1995, p. 1674) and greater emphasis on process value alongside outcomes.

In recent decades, participatory methods have been increasingly employed within disaster research, as both global initiatives (such as the Sendai Framework for Disaster Risk Reduction) and focussed case studies demonstrate their effectiveness in understanding the risks that communities face. However, ensuring true participation remains challenging (Mercer et al. 2008). Moreover, defining “true” participation is complicated by the wealth of methods available and the different levels of community participation they involve. Arnstein (1969), studying citizen involvement in planning processes in the USA, designed a typology of citizen participation with levels visualized as rungs of a ladder. McCall and Peters-Guarin (2012) adapt Arnstein’s ladder to disaster risk reduction (DRR) research to categorize participation intensities involved in common disciplinary research methods. Unfortunately, the lowest rung, *Exploitation*, is very common (Van Niekerk and Annandale 2013). Disaster researchers wishing to work ethically can choose methods with greater community involvement (from *Information sharing* to *Transformation*, where community members self-mobilize and invite outsiders to assist with research). Although the practices described in this paper differ from established participatory methods in disaster research (e.g., McCall & Peters-Guarin in Wisner et al. 2012), their similar intensities of community involvement allow the latter to serve as a comparative framework to evaluate the degree of community participation arising from this study’s methods.

This paper describes part of a research project conducted in 2021–2024 with people in communities on the flanks of an active volcano, Volcán de Fuego (hereafter “Fuego”) in Guatemala. Fuego is persistently active at a low level. It is also capable of major explosive eruptions (e.g., 10th – 23rd October 1974, 3rd June 2018) whose hazards have caused disaster for people in these communities. Eruptions in the second half of the 20th century affected agricultural livelihoods and contributed to emigration from communities on Fuego’s south-west flanks (Naismith et al. 2020). People who lived through these eruptions remember impacts vividly and include their experiential knowledge in their responses to recent eruptive crises (Naismith et al., 2024). However, many of these people have been persistently marginalized by the Guatemalan state and their experiences of past eruptions have not been told. This study was motivated by the prominence of these eruptions in these now-older residents’ collective memory and the desire to represent their experiences. The study aims to explore the potential of illustration to investigate experiences of volcanic disaster. Guiding research questions are: *(How) can illustration be used as a tool for exploring memories of volcanic disaster?* And *(How) can illustration be used to share these memories with at-risk people who have not experienced such disaster?* This project builds on previous studies that use creative methods of representation to deepen dialogue with participants:

When participants can ‘see’ representations of themselves in a visual creative work, they are more … invested in providing feedback because it holds greater personal significance, which opens spaces for dialogue that can lead the researcher to uncover deeper insights. Tatham-Fashanu (2023), as cited in Sou (2023), p. 328.

The paper continues by reviewing arts-based methods in disaster research, introducing zines and illustration as forms of representation and as potential research tools (**Arts-based methods to explore disaster response and recovery)**. **Process (methods & findings)** details the non-linear stages of the research process, including story collection, illustration, and consultation. **Zine design** outlines creative decisions, while **Discussion** evaluates project design and execution against existing literature, debating challenges and successes. **Conclusions** summarizes findings and suggests future methodological refinement.

## Arts-based methods to explore disaster response and recovery

Is disaster research methodologically stagnant? Schumann et al. (2019) suggest so, citing the tendency of disaster studies to cling to orthodox data-gathering methods such as interviews, surveys, and focus groups. In fact, many innovative arts-base methods to explore disaster are emerging, including photovoice (Schumann et al. 2019), music-enhanced interviews (Marsh et al. 2020), comics (Sou et al. 2021), poetry (Miller and Brockie 2015), graphic novels (Nalla et al. 2022), and interview-based zine making (Valli 2021). These authors prioritize ethical research by valuing participants’ individual experience and input in the research process. Arts-based methods in disaster research can help people reprocess trauma (Huss et al. 2016), diversify survivor narratives beyond homogenous accounts of “disaster victims” (Sou and Hall 2023), democratize the research process, and allow researchers to work more creatively (Sou et al. 2021). This shift in approach parallels advocacy for community-based disaster risk reduction (CBDRR), as researchers argue for a shift from paternalistic, top-down DRR approaches to those that require authorities to directly engage with at-risk people. Stone et al. (2014) examine the *vigía* network of Tungurahua volcano (Ecuador) to show how scientist-community collaboration can enhance risk mitigation, hazard monitoring, and community capacity. Paton et al. (2022) identify the need to include social and cultural processes in the development of CBDRR. Through case study, the authors envision how song and art – as representations of social-environmental contexts – could be used to develop DRR beliefs and actions, although they suggest that the visual arts may face greater accessibility challenges than music (Paton et al. 2022).

Zines (pronounced like “maga-*zine*”) are small-circulation DIY amateur publications, created by fans of a subject to connect with other devotees (Triggs 2006). Zines originate in 1930s science fiction fandom, when enthusiasts would create and self-publish fan magazines (hence “fan-zine” or “zine”) to fill the gaps between official releases (Coppa, 2006). Because their format allows creators to “make your own culture and stop consuming that which is made for you” (Duncombe, 1997, p. 2), zines have been a favoured means of expression among various counterculture communities in the 20th century, including the New York queer scene, 1970s punks, and Riot Girrrls (Triggs 2006; Romano 2023). Zines have endured as a form of communication beyond their pre-internet origins (Romano 2023). Rather, the internet has allowed zines and their communities to prosper in both digital and physical form. Perhaps this is because their original appeal remains: to connect a small number of like-minded people through sharing information and opinions in an idiosyncratic visual style. Zine-makers share a motivation to self-publish with avant-garde artists of the 20th century: “the desire to share ideas, ideology, art, etc., … otherwise underrepresented” (Gardner 2023, p. 4); thus as tangible artifacts produced by and for marginalized communities, zines hold both scholarly and societal significance. Zines also provide an alternative form of memory-making in response to trauma (Cooper, 2024). Taken with their accessibility and ease of distribution, zines offer an intriguing possibility for using the visual arts to explore experiences of disaster and facilitate development of DRR beliefs and actions.

To share their own positionality regarding zines and zine culture, zine scholars typically narrate their personal connection with zine making (Legendre 2023). I have been a practicing artist for many years but discovered zines more recently through local culture in Bristol. I made zines throughout 2020 in the lockdowns of the COVID pandemic. Making zines gave shape to this liminal time (Cooper, 2024). Furthermore, their materiality took on “a new affective dimension during lockdown” (Cooper, 2024): as life suddenly moved online, their tangibility grew attractive. This response echoes Piepmeier’s perspective of zines as ‘embodied communities’: “In a world where more and more of us spend all day at our computers, zines reconnect us to our bodies and to other human beings” (Piepmeier 2009, p. 58). Zines allowed me to reconnect to the outside and to other people while lockdown inhibited travel and in-person connections (e.g., a zine of a neighbourhood “treasure hunt” later published in my community magazine (Up Our Street 2021); a fantasy map of my PhD, shared with my university’s blog for doctoral students (BDC 2020)). I created zines to creatively explore volcano eruptive history and mythology^1^. These experiences provided my personal motivations for exploring local people’s experiences of Fuego volcano through zine-making and illustration.

The methods of this paper and of studies cited require considerable time and effort. Some researchers consider these methods a form of resistance against the demands of modern academia – including advocates of “slow scholarship”, a feminist movement arguing that quality scholarship must have unhurried time to ripen (Mountz et al. 2015). Studies exploring disaster through arts-based methods generally require researchers to build trust with at-risk people over time (e.g., a year in Puerto Rico before/after Hurricane María (Sou 2023); three years in three Indian states affected by floods (Nalla et al. 2022)). I have worked with people in communities around Fuego for seven years (2017–2024). I have developed trusting relationships with many people, which motivated our shared desire to represent their stories in their own words and my illustrations.

### Process (methods & findings)

**Fig. 1:**
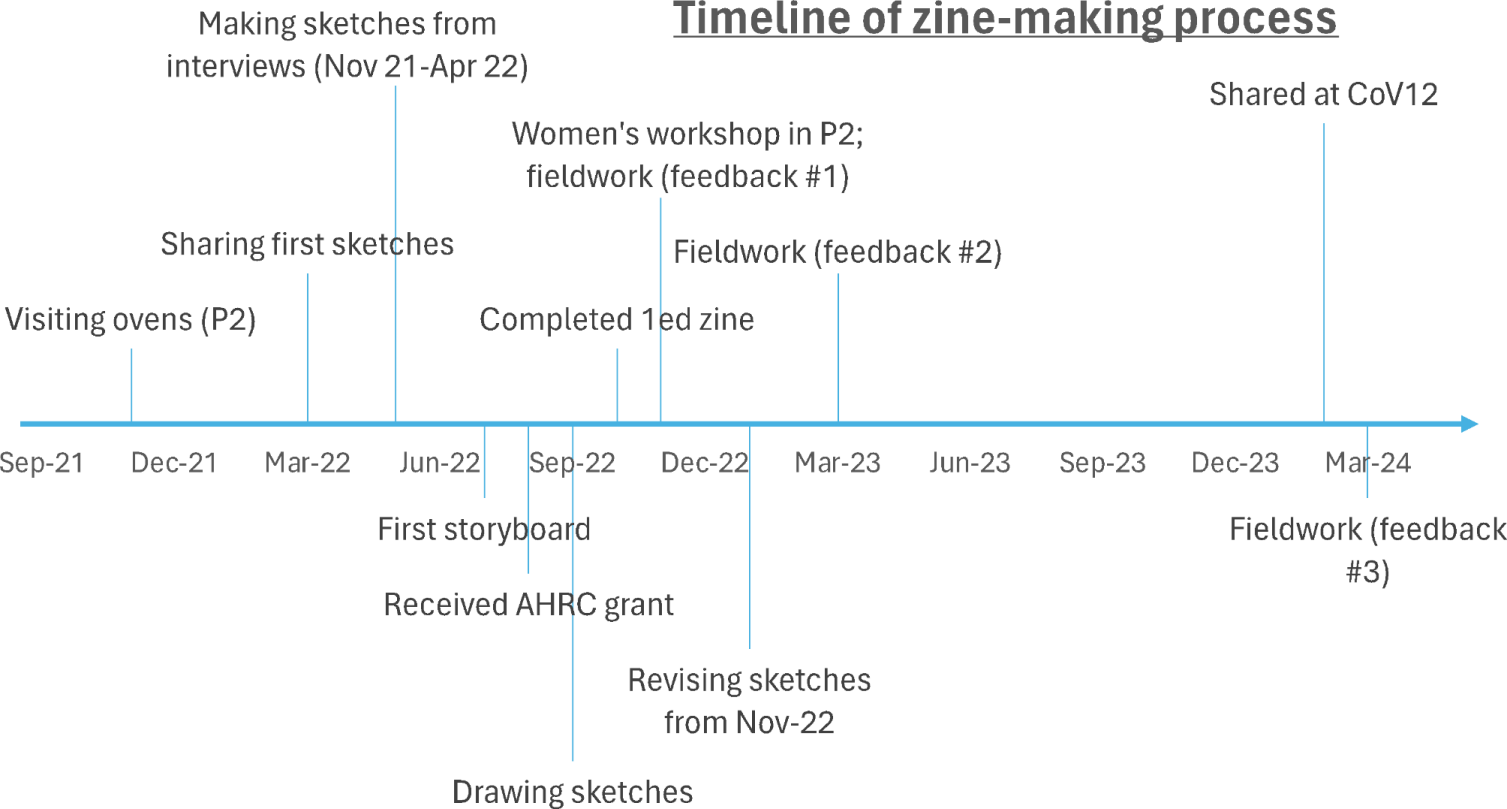
A visual timeline of the research process

Figure 1 chronicles key stages in this study. Although the zine includes some words from interviews in 2019, the formal timeline begins in November 2021 with the first period of fieldwork that focussed solely on people’s experiences of Fuego’s eruptions in the second half of the 20th century.

The zine tells the story of impacts of Fuego’s eruptions in the second half of the 20th century through the voices of local people. These events are also described through interview analysis in Naismith et al. (2024). In interviews and conversations in 2019–2024, older people shared how these eruptions negatively affected their lives and livelihood outcomes. They felt continually marginalized by the Guatemalan government, whom they accused of failing to address persistent impacts of Fuego’s activity (e.g., building bridges for people to cross rivers where volcanic flows descend). They expressed a desire to permanently record their experiences and to share them with younger people in their communities, who have not lived through a series of large eruptions comparable to those in the second half of the 20th century. We discussed ways to record and share these testimonies. While some community visits organically became storytelling sessions^2^, older residents also wanted a tangible document to carry their voices both within and outside their community. Several people suggested a book, but this proposal held challenges of varying literacy levels and limited time or resources. I proposed an illustrated zine as an accessible alternative – being easy to reproduce and disseminate, inexpensive, visually appealing, and not text-heavy – that was appropriate for their intentions and for the environment of Fuego. It is also an art form that I practice and enjoy (see **Arts-based methods to explore disaster response and recovery).** When I proposed a zine, describing it as a “simple book with drawings” where we could document their stories through conversations and illustration, people embraced the idea.

**Fig. 2:**
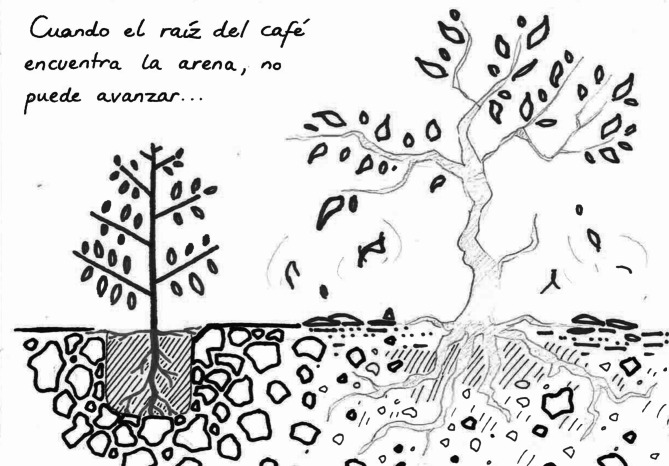
A sketch I made for a participant to illustrate his experiences of the effects of volcanic ash on coffee. The text translates from Spanish as, “When the root of the coffee meets the sand, it cannot go further”

Although art in disaster research is not new (see **Arts-based methods to explore disaster response and recovery**), this project was unusual in that I assumed the roles of both researcher and artist. One could theoretically make an unlimited number of illustrations about Fuego; in reality, time was limited for both me and participants. Therefore, we had to prioritize which stories to document. I made the first sketches for this project in 2021–2022, illustrating memories from interviews with older people living in communities on Fuego’s south-west flanks, principally the communities of Morelia, Panimaché Dos, and Panimaché Uno (Naismith et al., 2024). I drew for people with whom I could arrange another meeting to ensure opportunity for feedback (like consultations described by Tatham-Fashanu (2023) and Sou (2023)). For example, a sketch of a coffee plant struggling to grow in tephra deposited by eruptions in the 1960s and 1970s illustrated the experiences of a farmer living near Morelia (Fig. 2). While these exchanges were largely positive (pgs. 10–11), the time they required coupled with the demands of other research meant I was unable to organize group consultations with people about what should be recorded in the zine. These constraints and my dual researcher-artist role limited community participation in the zine’s development, giving me significant control over both creative and research aspects (see **Discussion**).

In the small number of participant consultations I held in early 2022, drawings I had made of their stories met with strongly positive feedback. When presented with the sketch, people expressed many emotions about seeing their experience represented in a drawing. They then spoke about their memory in greater depth. This is the exchange I had with the participant whose experiences inspired Fig. 2:

Participant (Morelia): And how excellent, that you have elaborated this, because if one can see that what we have explained is fully understood, because that is our reality. We have to dig a hole. And fill it with brushwood, with organic material that other trees throw away, to fill the hole here. We put the [coffee] bush in and it grows and there we fertilize, there we water. And there we harvest. But once the bush has already consumed what is here, we must replenish that ….

An older man from Morelia recalled a prawn falling from the sky into his hands during an apocalyptic eruption (**SM-2**, pg. 10). I heard multiple versions of this incident, with some people recounting a fish instead. Every narrator connected the incident to a major tephra-producing eruption and asked me if it implied that the volcano was connected to the sea. The prawn’s recurring appearance in local memory implicated it as an important element in stories that people told of Fuego, and gave another opportunity to explore how illustration could deepen dialogue about these eruptions. When given the drawing, the man expressed joy and elaborated on his memory:

Participant (Morelia): Yes, well. Here are other little houses, see. And so many little things … And look, all this. These other houses. You did well [gesturing to the drawing].

Researcher: Ah, yes, you like it? … This is Morelia I drew, because look, you can see the coffee beans^3^. So I drew Morelia, and the road to Panimaché Dos.

Participant: Yes, yes, yes, yes, yes. This is the road.

Researcher: That’s it, that’s it. So just going up the road from Morelia to Panimaché Dos.

Participant: For Panimaché Dos it is this. Before. Before this was Panimaché Uno. Before this was Panimaché Uno. But all the people fled because of the volcano.

Despite my extensive previous work in these communities, these consultations had a uniquely rich quality. People’s responsiveness to seeing their experiences represented by illustration mirrored the findings of Tatham-Fashanu (2023) (quoted in the **Introduction**) and moved me inexpressibly. Creating illustrations for people at Fuego, and sharing it with them, seemed to “offer … a direct testimony about the world which surrounded other people at other times … in [a] way … more precise and richer than literature” (Berger 1972, p. 1). Reflecting on the project after its end, I have attempted to articulate this quality (see **Discussion**).

My next opportunity for fieldwork was in November 2022. Because previous fieldwork had demonstrated the large time requirement for both illustration and consultation, but I could not consult directly with people in summer 2022, I developed an alternative method. I illustrated key themes and phrases from interviews with older people; the themes’ recurrence indicating their significance in collective memory. The dataset comprised 45 interviews with 57 people in two study periods (Feb – Apr 2019, Nov 2021 – Apr 2022); full details appear in Naismith et al. (2024). Interviews followed ethical procedures, including obtaining participant consent, ensuring confidentiality, and sharing contact details with participants. This study was conducted entirely in Spanish; all quotations from Fuego residents have been translated to English by me specifically for this paper. To identify key phrases, I ran text queries in NVivo12. Query returns included: “*todo se oscureció*” (“everything went dark”, referring to the sky darkening with ash) and “*después vinieron los lahares*” (“afterwards came the lahars”, recalling the mudflows that descended Fuego’s ravines following large eruptions). Major themes were identified by iterative coding sessions, following a flexible approach outlined by Deterding and Waters (2021). These themes included migration around Fuego and social events that coincided with eruptions (e.g., Fuego interrupting a church inauguration). Although some stories of eruptions contained fantastical elements (e.g., the falling prawn), people did not generally tell or know of legends about Fuego. Through this coding process, key phrases and themes that were collectively significant and richly sensory – and therefore suitable for illustration – began to coalesce. I made a storyboard for the zine in July 2022 (see **SM-1**) and applied for seed funding from an arts council grant. I proposed to use illustrations in consultations with participants whose memories were illustrated to invite suggestions and direct the development of the zine in an iterative and collaborative process (as in Sou and Hall 2023). I printed the illustrations as a booklet in October 2022. This format afforded flexibility to share illustrations as either single or multiple pages.

Fieldwork in November 2022 comprised several individual and group consultations and two participatory workshops. I invited someone from the local community to facilitate for their knowledge of local cultural context and their familiar presence that would give people confidence to share. A facilitator from Panimaché Uno assisted in some consultations, and another from Panimaché Dos facilitated that community’s workshop. Consultations took place in Morelia, Panimaché Dos, and Panimaché Uno. I arranged consultations either by telephone or by visiting in person. Most people were pleased to be contacted and agreed to participate. However, participants in November had more mixed feedback than in March. During a group consultation in Panimaché Uno, three men who had previously suggested documenting their experiences as a book briefly looked at my illustrations of their memories before eagerly returning to sharing their stories. I had several similar interactions during this fieldwork. Some consultations did produce deeper dialogue. A woman in Panimaché Dos initially found little to revise in my illustration of her memory:

Participant (Panimaché Dos): I don’t know what you want. More, more information.

Facilitator: More information.

Researcher: Or – for example, what we were talking about … does it look good, or is something missing? Because I imagine … this is only from my imagination. So it could be that there are things that I have done, that maybe do not appear exactly. Like this, “Llovió con ceniza”, right? And it was not ‘ceniza’, but ‘arena’.

Daughter of participant: She wants you to help her, so that everything is right.

Participant: Ah, to correct things.

Researcher: Mm-hm. Because certainly there are errors. This came out –.

Participant: No, almost – almost everything is right. Mm-hm.

However, she later dwelt on my illustration of another of her memories, an eruption that interrupted a church inauguration (**SM-2**, pg. 8). Tracing the illustration with her finger, she elaborated:

Participant (Panimaché Dos): The saucepans of food, there they are.

Researcher: Ah, yes. What are they, what were they cooking?

Participant: When it was about the sand, it was beef broth. […] I was nine years old.

Daughter: You are sixty-seven now.

Participant: And now I am sixty-eight. […] I was nine years old when that happened. I remember that with my cousin [we went there]. That’s why they ran away like it was hail, but what a big hail of sand! And everyone was in the tent. The brothers with bars like that, because as … you know the tent, the tarp. It was sinking.

**Fig. 3:**
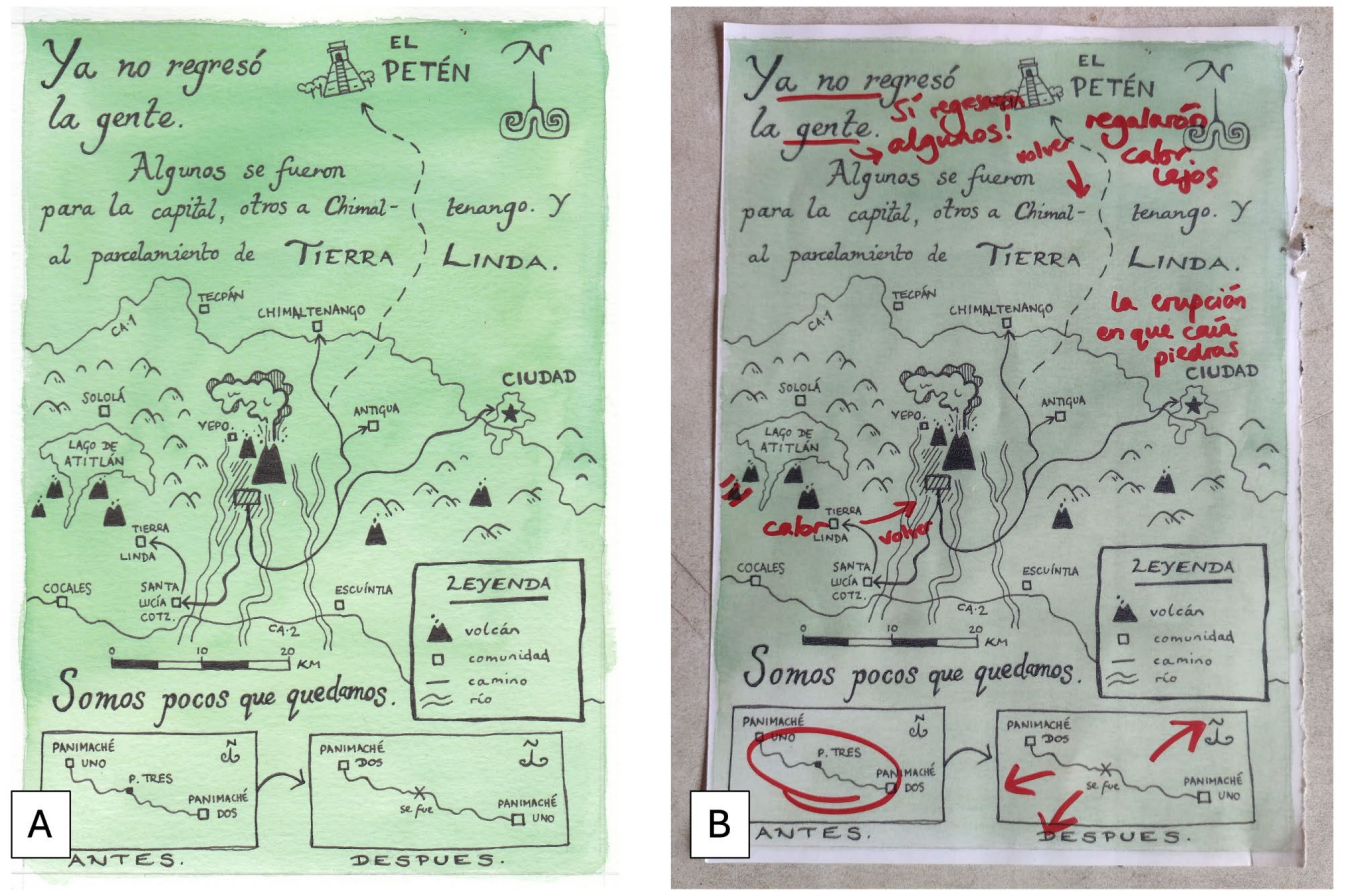
Example illustrations from fieldwork in November 2022 showing (**a**) a single illustration from the zine; (**b**) the same illustration annotated during a consultation with participants in Panimaché Uno. I had previously spoken with these people in interview where they recalled migration from Fuego (the subject of this page)

Consultations where I shared one or two illustrations provoked the most dialogue. Participants used the illustration to talk about their memory and link it to others, and when I asked for feedback on the illustration, made comments or suggestions which I noted down on the page (Fig. 3). After consultations I recorded my observations and reflections through field notes and audio recording.

**Fig. 4:**
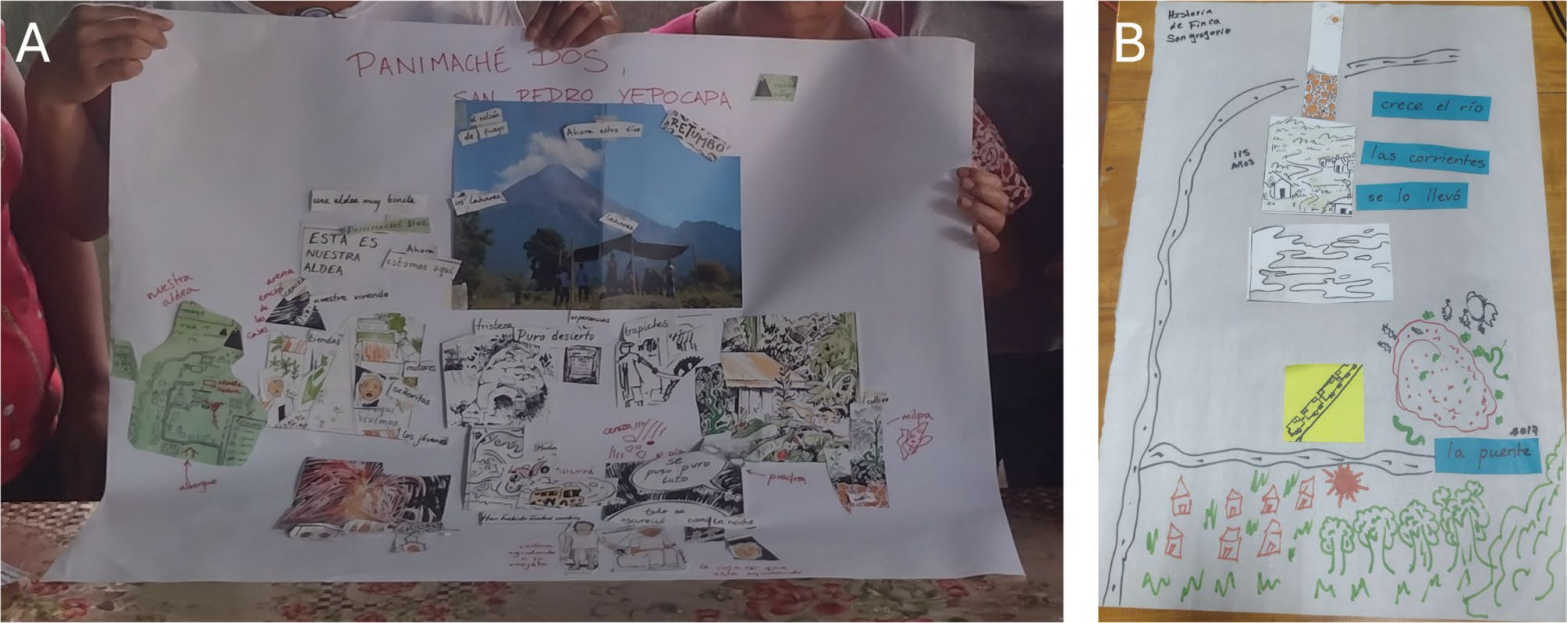
Outputs of participatory activities held in November 2022 with (**a**) women in Panimaché Dos and (**b**) women in Siquinalá. Output (**a**) was developed collectively by participants, while (**b**) was created individually by a participant from Finca San Gregorio (a community on the coastal plain south of Fuego regularly affected by floods swollen by sediment from volcanic activity) and shared with the group

I held two participatory workshop activities in November 2022. The first was within a broader workshop within the GCRF-funded project *Ixchel* for women around Fuego organized by Drs Teresa Armijos Burneo and Cristina Sala Valdés in the town of Siquinalá. The second was with a group of women leaders in Panimaché Dos. The workshop activity comprised three stages. First, I would open the space by introducing myself and inviting everyone to do the same, and I would explain that the purpose of this activity was to come together to exchange experiences of living beside Fuego. Second, I would share a large piece of paper with the prompt, “*Tell me about your community and its history with Fuego*”. Participants were offered paper, pens, and cut-up pieces of illustrations and words from the zine, given time to create their stories, then share with others. Finally, the group would come together to discuss what they had learned. In practice, the activity worked differently each time. In Panimaché Dos, the women worked together on a single page that included their own experiences and elders’ stories of the community’s past (Fig. 4a). In Siquinalá, women from different communities took turns to tell their individual stories, using words and illustrations from the zine as prompts (Fig. 4b). Although the workshops were highly interactive, levels of engagement varied between individuals. I developed the activity design while in the field, partly because of my concerns about the level of community involvement in the zine-making process (see **Discussion**).

During my next field visit in March 2023, I aimed to share a second edition of the zine, which incorporated participant feedback through revised and new illustrations that I had made since November. However, this visit proved the most challenging so far. Many participants were not available to meet with me during my short (one week) visit, as it coincided with the peak of sugar-cane harvest season when many older men were working in the fields. To use my time well, and to explore my second research question, I arranged to meet some people in Panimaché Dos who agreed to evaluate the zine. Although these people had not participated in earlier consultations, they represented at-risk people that older residents had earlier identified as among the intended audience of their documented stories. One woman who had lived in Panimaché Dos for decades, and whose husband I had talked with extensively about Fuego, said: *“Esta historia no es de nosotros”* (“This story is not ours”). This striking comment encouraged me to reflect critically on the limitations and benefits of zine-making in sharing memories with at-risk people who have not experienced disaster (see **Discussion**).

I had a final opportunity to evaluate the zine in March 2024 on a visit to Panimaché Uno. I invited community members to a meeting in the Fuego observatory. While some briefly admired the zine then returned to sharing other stories, one woman commented:

*Participant (Panimaché Uno)*: What is in the books … for me, it seems good. I like it. […] I grew up in the other Panimaché [Dos]. There were also bakeries, there were […] other things. Very nice, right. And looking at these books, you remember what your grandparents told you. […] And the part about this village, well, I didn’t know anything about it. And seeing this book, one already… sees what has gone before. And everything was destroyed. Because now, you don’t see any of this anymore. Bakery and so on, there is nothing anymore.

**Fig. 5:**
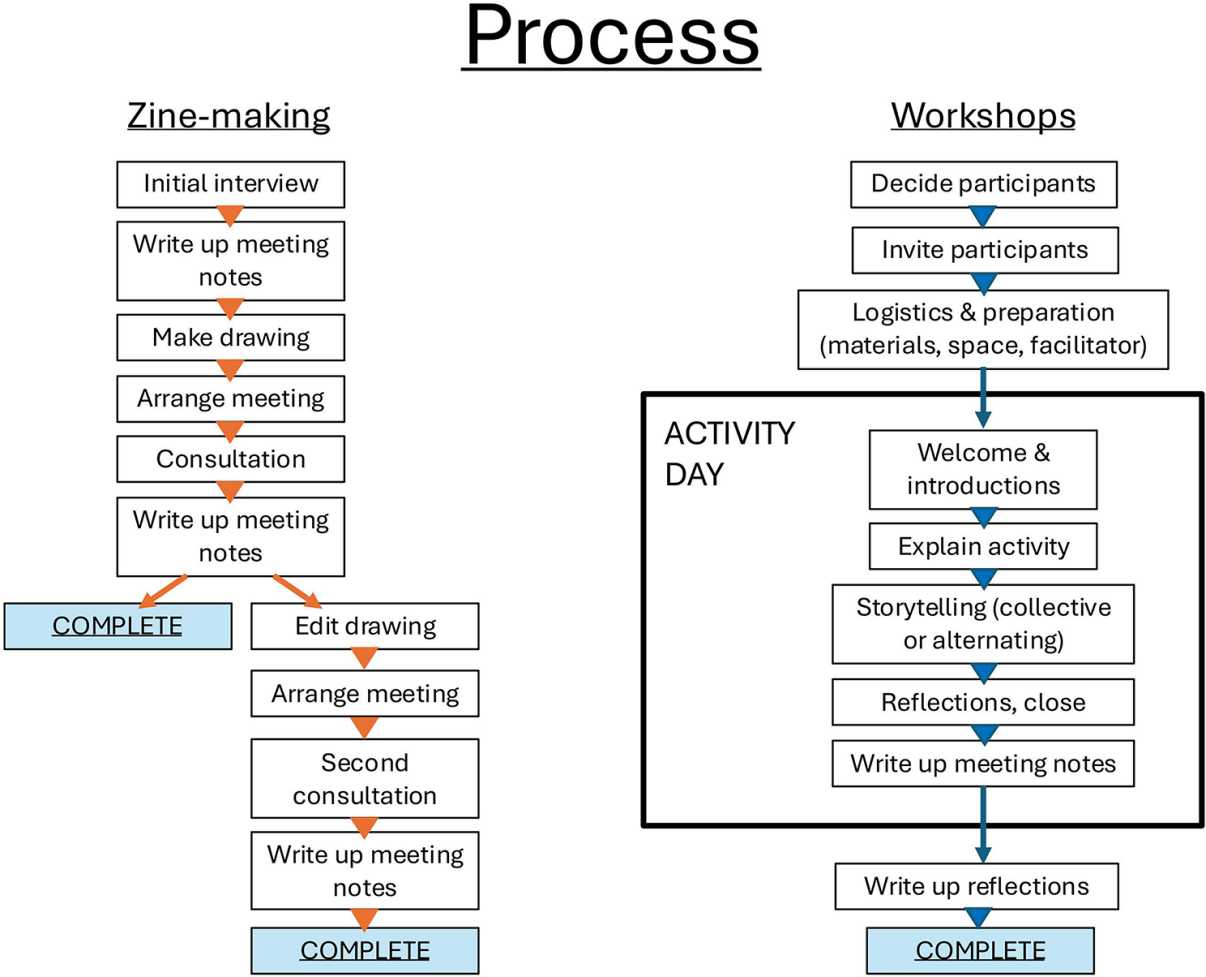
Diagram of the research processes I followed in this project, describing both consultations on illustrations and group participatory activities. Both processes involve considerable investment of time and resources. The challenges and successes of translating this design into practice appear in **Discussion**

Figure 5 outlines the extensive time commitment required for the elements (including consultations and workshops) comprising my research process. “COMPLETE” represents a suitable pause point for a research strand rather than an ending. The following section, **Zine design**, briefly explores the decisions involved in making the zine. **Discussion** critically evaluates this approach and the challenges I encountered and offers guidance for other researchers considering illustration or zine-making as forms of representation to explore lived experience of disaster.

## Zine design

The zine was assembled from sketches, drawings, and illustrations I made in 2021–2023 (see **Process (Methods & findings))**. Some pieces were included unrevised in the final version, while others (e.g., pgs. 8 and 29) were revised following consultations. Because of my strong relationship with people at Fuego, our shared desire to tell their stories of its eruptions causing disaster, and my experience with making zines, I wanted to create a visually appealing work where illustrations accompanied direct quotations of people’s lived experience to share their unfiltered voices. The stories people had shared with me across years at Fuego afforded the possibility of creating a work fusing eruptive history and mythology and provide an alternative form of memory-making in response to trauma (see **Arts-based methods to explore disaster response and recovery**). Although I initially considered a zine with a simple fold-out format, people had shared so many stories with me that this format felt limiting, even after the process of selecting quotations and prioritizing stories. The final version of the zine is 34 pages. These pages contain a fraction of stories around Fuego, and yet they required enormous effort and time. The zine was intended to recognize the wealth and vividness of older people’s stories and their generosity in sharing them, as well a way to capture an environment and the lives within it that are marginalized and so rarely seen. The zine design choices were largely driven by my experiences, and the following section discusses the ethics and tensions of this approach.

## Discussion

This study set out to explore two questions: *(How) can illustration be used as a tool for exploring memories of volcanic disaster?* And *(How) can illustration be used to share these memories with at-risk people who have not experienced such disaster?* Drawing on existing literature, I critically evaluate how well my research addressed these questions. The evaluation reveals challenges and successes in two key themes: participation and representation.

**Fig. 6:**
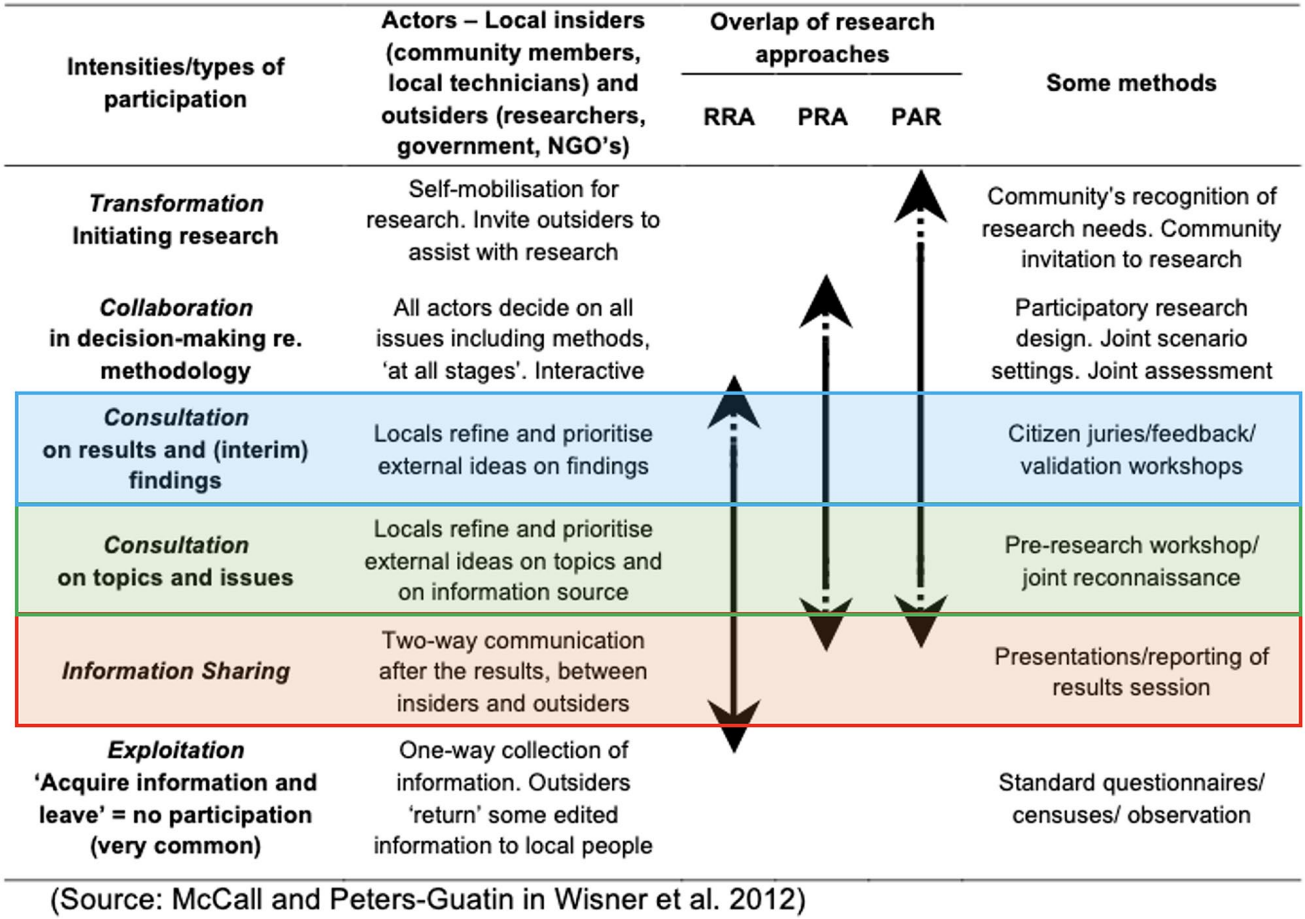
Intensities of participation in disaster research (from Wisner et al. (2012) in Van Niekerk and Annandale (2013, p. 163). Coloured boxes (my addition) indicate the intensities of participation invited by my methods

Throughout this study, I questioned how participatory my process truly was. The flexibility of participatory research complicates evaluation of this question, as community involvement varies even within single methods. I chose to evaluate community participation in my process by measuring it against existing frameworks. Arnstein’s (1969) ladder of citizen participation has been adapted to disaster research to visualize different ‘intensities’ of participation (Van Niekerk and Annandale 2013, pg. 163); I have modified that figure to show the methods in my process and my evaluation of the intensity of participation each method invited (Fig. 6). At the lowest intensity, *Information sharing* involved presenting several pages or an entire zine to people and inviting their feedback during field visits (e.g., group consultation, pg. 6; orange box, Fig. 6). *Consultation on topics and issues* involved sharing an illustration with individuals to prompt deeper dialogue about their memories (e.g., consultations, pgs. 5-6; green box, Fig. 6). Workshop activities in November 2022 reached between *Consultation on results and interim findings* and *Collaboration*: participants worked interactively and decided research direction (pg. 8; blue box, Fig. 6). In the Panimaché Dos workshop, the group worked together to create a collective story of the community that included both their lived experiences and stories heard from elders. In the Siquinalá workshop, women alternated as storytellers and listeners, storytellers using zine materials to narrate their own experiences of Fuego. These activities hold promise for communicating volcanic disaster with at-risk people without similar lived experience by sharing stories between communities (Siquinalá) or across generations (Panimaché Dos). Participants’ ready use of illustrations demonstrate how cultural forms (art, stories, rituals) help people express their experiences within their community’s context and understanding (Huss et al. 2016).

**Fig. 7:**
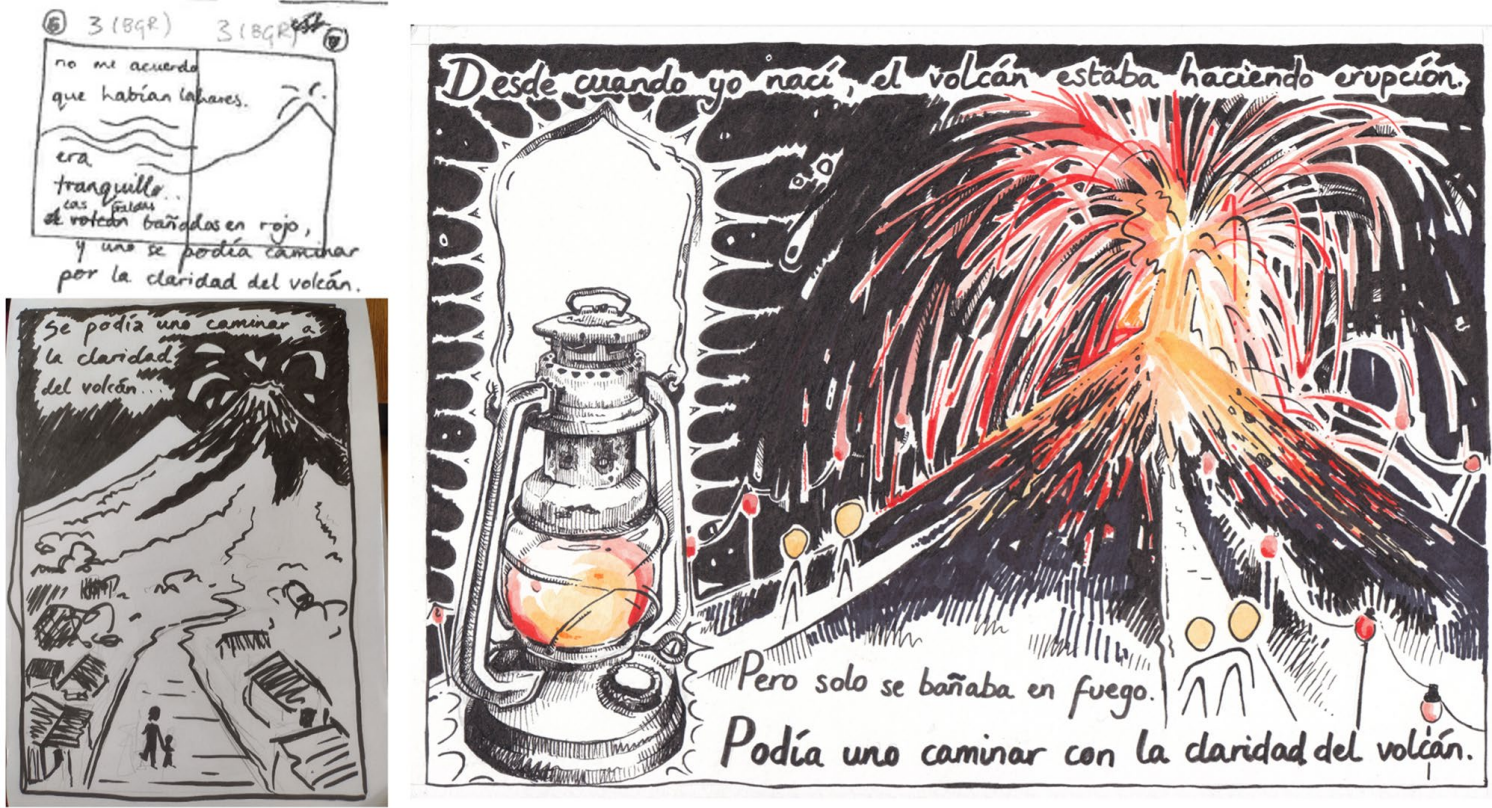
Development of one of the zine pages, from (top left) first doodle in storyboard to (bottom left) more evolved sketch to (right) final version in ink and watercolour. The text translates as, “Ever since I was born, the volcano was erupting. But it only bathed itself in fire. One could walk by the light of the volcano”

Examining consultation processes offers another way to evaluate community participation, as these are commonly detailed in participatory research. Sou (2023) describes her consultation process for *After María*: “I invited participants to give feedback … People felt comfortable directing, critiquing, and suggesting amendments and ideas because they could literally see their environments” (Sou 2023, pp. 327–328). Similarly, Tatham-Fashanu (2023) describes actively co-creating comics with participants. In my consultations, participants’ feedback was more equivocal: some people readily made suggestions, while others offered only approval; rarely did people critique the work. What produced these differences? Cultural unfamiliarity does not seem significant: neither Tatham-Fashanu nor Sou worked with participants who were deeply familiar with comics^4^, and both argue that visual methods are universally accessible. However, the quality of art presented for consultation may have contributed to these differences. Tatham-Fashanu’s comics feature stick figures, which she describes as a simple and comprehensible visual language; Sou shared rough pencil sketches. My more detailed illustrations (Fig. 7**)** may have limited participants’ feedback because they were satisfied with the existing illustration, because our personal relationship meant they felt awkward about critiquing my drawings, or because they were unmotivated to engage because they did not see an opportunity to “deeply influence” the research (Sou et al. 2021). Examining my methods revealed tensions between my roles as researcher and artist, with zine-making and illustration inviting different levels of participation than initially intended. These tensions complicated my research objectives.

Zine-making can achieve high community involvement when researchers invite participants to drive the process (Valli 2021). Even solo-created zines can invite community engagement through their unfinished quality: Piepmeier (2009) illuminates how the ‘scrappy messiness’ of zines starts conversations between creator and reader. By contrast, a drawing is a static image whose value depends on its fixedness (Berger 1976). The finality of my artwork may have discouraged participants from suggesting changes. However, when drawings were cut up for workshop activities, their finished quality dissolved and participants freely shared their volcano stories through them. This difference in participation invited by zine-making and illustration mirror tensions between my roles as researcher and as artist, but also offer different ways to address my research questions. My research aim was to amplify the voices of marginalized people – and community-led participatory methods yield best results for understanding communities’ disaster risk profiles (van Niekerk and Annandale 2013). However, artists spark dialogue by sharing their vision, making their role central: “we are always looking at the relation between things and ourselves” (Berger 1972). My dual role raised questions: were participants collaborators or just critics? Combining zine-making and illustration made this hard to define. Each method promised ways to address my research questions: participatory workshops provided materials and space for people to creatively share stories of disaster with others, while in consultations, illustrations provoked deeper dialogue with individuals about their memories. In future, combining these methods to communicate disaster experiences might address the ambiguity of participant involvement by inviting more collaboration at outset (e.g., initial group consultations of zine content) or by limiting the researcher’s role (e.g., by engaging a separate artist). However, the desire to work creatively motivates many researchers to use these methods (Sou et al. 2021). Although researchers wishing to use creative practices must match their skills with their research context, not just enthusiasm (Miller and Brockie 2015), participants’ responses in early consultations suggest it was appropriate to explore my artistic skill at Fuego.

Zine-making and illustration offer different means of representation as well as levels of participation. Representation encompasses both portrayal and being portrayed. Drawing is portrayal, an attempt to discover or describe how one sees things (Berger 1972). While zines can involve representation in both senses, their value often lies in giving marginalized communities means to portray themselves on their own terms (Gardner 2023; this study, pg. 3). The difficulty of answering, “Whose vision is represented in the zine?” reveals tensions between my artistic choices and community input. The comment, “This story is not ours” (zine evaluation in March 2023) indicates that this woman did not feel connection to the zine and the stories it represented and suggests that representation alone is insufficient to communicate volcanic risk to at-risk populations. This interaction revealed a key limitation: with no zine narrators present, there was no direct dialogue between disaster survivors and at-risk populations. Research shows that effective risk communication to vulnerable populations requires continuous local involvement (Mercer et al. 2008), so these limitations could be addressed by ensuring zine narrators are present in such interactions. Older people at Fuego wished to share their stories with younger generations to inform them about Fuego’s risks and, where possible, take preventative action (see **SM-2**, pgs. 26–27). Extensive research explores “boundary objects”: outputs from participatory arts-based methods that bridge social worlds and inspire action:

…boundary objects are often material artworks, collaboratively made by participants living in marginalized situations, whose faces and voices are unseen and unknown. The objects are often displayed in public spaces or sessions to exchange knowledge with stakeholders, and create empathy about experiential knowledge. Groot and Abma (2021), p. 2.

Boundary objects enable genuine connection but require researchers to create spaces for equitable dialogue (Groot and Abma 2021). The Siquinalá workshop, where storytellers used illustrations to share their stories, exemplifies such a space. When I shared the zine with another community near Fuego in March 2023, it received an enthusiastic response, with residents requesting a similar project to document their own volcanic experiences. Their desire to share stories with younger generations echoed the original motivations behind this project, indicating the potential of zines and illustrations as boundary objects for communicating disaster narratives. Such work demands ethical care. The interaction above highlights ethical pitfalls in practice, particularly the absence of dialogue and uneven power dynamics in representation. While uncomfortable, the interaction illuminated elements essential for implementation: facilitating spaces for open dialogue and ensuring narrator presence.

Community participation varied across the research process partly due to time restrictions: after the first fieldwork period, my visits at Fuego were short and my time shared with other projects. This made it difficult to contact participants, organize meetings, and build a sense of continuity across the study duration. Local facilitators helped immensely but were not always available due to travel difficulties and family concerns. While these ordinary logistical challenges are hard to avoid in rural Guatemala, they inevitably mean that I afforded myself a great deal of power over the research process. The elements required for community-led participatory research – trusting long-term relationships, an evolving process, inclusion of many stakeholders – are also challenging to implement in the current funding landscape. Grants are often awarded short-term and based on presumed impacts, while: “the nature of arts-based inquiry does not allow to know all the details of the process, conclusions or possible impact in advance” (Coemans and Hannes 2017, p. 42). The AHRC grant supporting this study allowed the project process and outcomes to evolve in vivo and invited grant holders to apply for follow-on funding for their projects: elements supporting self-evaluation and longitudinal study. Funders wishing to support participatory research might consider how the grant conditions may support or inhibit the practices required to realize research objectives.

The challenges I encountered in community involvement and representation relate to a key struggle in arts-based participatory research: the “notion of empowerment” (Coemans and Hannes 2017), or the difficulty of translating ideals of community empowerment into practice. First, I had to acknowledge the study limitations: any empowerment people obtained would be constrained within the larger political, social, and economic insecurities of Guatemala: “one project alone [cannot] radically transform either the individual or the system within which they have to operate” (McKean 2006, p. 320, as cited in Coeman and Hannes 2017, p. 41). While representation of disaster experiences is vital, successful risk reduction at Fuego requires local institutional integration – one of the most challenging aspects of implementation (Few et al. 2022). Although I was cognizant of local power relations (essential for effective participatory DRR research (Mercer et al. 2008)), it was very difficult to include participants at all stages and to give a voice to everyone with whom I worked – not just the eloquent or confident. My control over the research and creative processes meant that I was often the decision-making authority, which brought ethical challenges. Some choices intended to maximize representation unintentionally reinforced that authority. For example, although the zine included participants’ memories quoted verbatim – “The goal was to present participants’ voices as raw as possible” (Valli 2021) – I ultimately decided which memories to include in the zine. While participatory research inherently involves some power imbalance (Few et al. 2022), I made several efforts to diversify participation and mitigate this imbalance. In the Panimaché Dos workshop and several consultations, I engaged a member of the community to facilitate. Although seven years working at Fuego have given me a rich understanding of local culture and language, I am still an outsider. The facilitators enriched conversations by adding knowledge of local context and bridging gaps in understanding, thus contributing to a trusting atmosphere and mitigating issues of researcher-participant power relations (Mercer et al. 2008; Le De et al. 2014). Workshops were designed so people used their voices to direct activity and tell their own stories. Meanwhile, I tried to include people’s voices in consultations through their narration of early sketches and later illustrations, and their opinions were sought to comment on and revise these illustrations. Drawings changed in response to feedback included pages 8 and 29, and drawings that were added included pages 10 and 13. Arts-based participatory methods can facilitate community empowerment through research process ownership (Valli 2021) or representation of individual experiences of disaster (Miller and Brockie 2015). While feedback suggests that the methods presented here require refinement, evidence for empowerment through representation emerged in some participant’s responses (see end of this section). These combined methods show promise for addressing both research questions. Future iterations could incorporate participant-led knowledge exchange sessions, utilizing zines as boundary objects for community narrative sharing. Experience suggests community appetite for such peer-to-peer risk communication forums (footnote 2).

Alongside challenges to community participation and representation, working as both artist and researcher offers unique advantages. While researchers have used various arts-based methods to understand multiple experiences of disaster (**Arts-based methods to explore disaster response and recovery**), illustration and zine-making remain underexplored despite their proven strengths in representation (Berger 1972; Gardner 2023). Both investigative and creative skill require reflection, practice, and contextual knowledge. Through my years working with people at Fuego and making art, I was uniquely able to bring my skills in both research and art to a context where I was knowledgeable, sensitive to trauma, and able to amplify people’s voices of disaster. While I am not aware of existing criteria for evaluating the quality and impact of illustrations for exploring disaster experience, prompts for a poetic approach provide a possible evaluative guide:

Critically, in evaluating the quality and impact of a poetic approach, Sparkes and Douglas (2007) suggest four guiding prompts to consider: aesthetic merit (e.g., are they artistically shaped, do they create evocative connections), their impact (e.g., do they affect the reader emotionally and intellectually, generate new questions, move people to action), ontological and educative authenticity (e.g., do they stimulate reflection from research participants and others) and, finally, a consideration of ethical questions (e.g., participants engaged in process). Miller and Brockie (2015), p. 106.

In one interview, a participant recalled admiring Fuego’s incandescent fire fountains at night in the years before its destructive eruptions in the 1960s and 1970s. I developed his testimony into a detailed illustration that included his description verbatim **(**Fig. 7**)**. When I gifted the participant this artwork in a later consultation, he expressed a mixture of joy and sadness, described how the paraffin lamp evoked memories of using candles in the darkness, and expressed a hope that the drawings could be shared with younger generations to inform them of Fuego’s history. This and other consultations (e.g., with participants quoted at close of **Process (methods & findings)** and **Discussion**) address the four considerations for poetic enquiry and give qualitative support for visual methods to encourage representation and deeper dialogue of disaster (Tatham-Fashanu 2023). Future iterations of these methods might involve or adapt the poetic approach above to evaluate their quality and impact more comprehensively, including their emotional significance.

Coeman & Hannes (2017) urge researchers using arts-based methods to reflect on both process and output. My dual researcher-artist perspective afforded me nuanced insights into both. In this study, the relationship between participation levels and empowerment proved non-linear: highly participatory activities did not guarantee engagement), while less participatory approaches sometimes achieved meaningful representation. This suggests that for researchers wishing to use arts-based methods with communities, the objective is not simply to ‘reach the top of the participatory ladder’ **(**Fig. 6**)**, but instead evaluate the different benefits that more and less participatory approaches offer. While consultations produced the zine, workshops created a rich space for storytelling by prioritizing process over outputs. Workshops also yielded unconventional co-created artifacts (see **SM-3**). This aligns with Valli’s (2021) argument that combining different levels of participation in research design can optimize research outcomes.

Although arts-based participatory methods offer new ways of processing traumatic memories (Huss et al. 2016), care must be taken to avoid retraumatizing participants. While participants found meaning in visual storytelling, and illustrations deepened our dialogue, depicting trauma raised ethical concerns of causing harm by reanimating their painful memories. I was uncertain whether an outsider illustrating such memories would be beneficial (in recording people’s lived experience) or detrimental (in recalling pain). When receiving illustrations of their memories, participants often expressed simultaneous pain and appreciation. My dual role as artist-researcher, together with my longstanding relationships with people at Fuego, allowed me to sensitively handle these intimate conversations. Early consultations in 2021–2022  suggested visual representation would be valuable, while my research experience helped create safe spaces for these discussions.

“Attention is the beginning of devotion” wrote the poet Mary Oliver (Oliver 2019, p. 5); she positions attention as life’s essential work. Paying attention twice was a unique advantage to my dual artist-researcher role. By listening to the stories people told of Fuego, I learned how they had experienced its eruptions and impacts. I documented these experiences to amplify their voices, to understand how their past experience influences current crisis response, and to preserve fading memories – most witnesses to these eruptions are in their 60s and 70s. Drawing complemented this research work as another means of understanding and preserving experiences at the edge of living memory. But drawing offered something more. Local people shared their memories of Fuego in vivid language, employing gestures and sound effects when describing climactic moments. In our dialogue there transpired a kind of magic: a participant narrating their experience could conjure a powerful image inside my head that I could translate to paper. My illustrations tried to convey this “seeing which comes before words and can never quite be covered by them” (Berger 1972, p. 1). Each illustration accompanied a verbatim quotation to honour the interaction that produced it. Although this approach invited less participant involvement, it showed the myriad power of illustration to represent disaster experience: looking as an act of choice, drawing to honour researcher-participant connection, and drawing to challenge disappearance. A drawing “…involves, derives from, and refers back to, much previous experience of looking. Within the instant of the sight of a tree is established a life-experience. This is how the act of drawing refuses the process of disappearances and proposes the simultaneity of a multitude of moments” (Berger 1976, pp. 43–44). Illustration to challenge disappearance suited a project based on lived experience of disaster among older people, while illustration from looking as an act of choice communicated to local people my continued interest in working with them and recording their experiences. Illustration to preserve the past and open conversations about participants’ life stories parallel other research using creative methods to sensitively discuss traumatic memories (Marsh et al. 2020). Likewise, a “zine positions [readers] as friends, equals, members of an embodied community who are part of a conversation with the zine maker” (Piepmeier 2009, p. 71). My dual researcher-artist role offered multiple means to connect with participants and demonstrate my commitment to record their stories. For researchers who have devoted time to working with at-risk communities, zine-making and illustration offer powerful ways to “pay attention” creatively to honour that connection.

Sou et al. (2021) advocate graphical illustration of research to encourage participant influence and to create accessible research products. For these reasons, and those expressed above, I undertook this difficult and rewarding project. This project benefited from expertise from many colleagues in Ixchel and deepened my existing relationships with people around Fuego. Guided by Coemans & Hannes’s (2017) call to explore both process and outputs, I share these insights for other disaster researchers considering using creative participatory methods:

1. **Consider control.** I held a large amount of control in research and creative processes of making the Fuego zine. In workshop activities, participants engaged strongly and directed the process; I also had to accept giving up control over research outputs. Varying levels of community participation offer different advantages. How much power do you (as the researcher) hold in your study design? How (much) will you invite participants to decide the research direction, and what would the process gain if you relinquished some power?
2. **Process or output?** Making the Fuego zine was focussed on creating a tangible output. Participatory activities focussed on the process of creating understanding through telling stories in a trusting environment. Other scholars note the different virtues of research design focussed on process and on output. Which parts of your study design are devoted to process and which to output(s)? Does the form of your representation change from process to output?
3. **Consider the investment and value of slow scholarship.** This paper summarizes a three-year project comprising copious notes, dozens of conversations, and multiple sketches, none of which will ever appear as a research output. I found the project slow, time-consuming, and intensely rewarding; it redoubled my connection with Fuego and its people. This project was made possible by the scope of Ixchel and the flexibility offered by the AHRC grant. Are you able to invest in a participatory project? What funding avenues can support your project?
4. **How many voices?** Despite my experience of working at Fuego, I struggled to give everyone a voice, from inviting the shy to speak to managing ‘protagonists’ who always spoke, sometimes over others. It is important to actively extend participation, while accepting that community engagement is often achieved through a few motivated individuals. I engaged a local facilitator and worked with existing community groups to address these issues. What methods will you employ to maximize representation of many voices? How can you work with motivated individuals to broaden participation?
5. **Consider cultural differences.** The ideas in this project are familiar to me through my research network and UK culture (specifically local culture in Bristol). By contrast, they sometimes met with confusion when I discussed them with people at Fuego. Some effort was then required to translate the ideas into language more familiar to people at Fuego. Dedicating some time to translation of culturally unfamiliar ideas is essential.
6. **Everybody needs to feel important.** Some powerful moments in this project came in individual consultations when I gifted a person an illustration of their personal memory. The moments held rich emotion for me and often for the participant. These encounters accord with other researchers’ reflections on the importance of recognizing people’s individual stories of disaster. Consider how your research design makes participants feel important.
7. **The importance of coming back.** Independent of their responses to the research process, people at Fuego were delighted when I returned. Whenever possible, it is important to come back: investing time in being together avoids some of the worst tendencies of exploitative research. Returning also honours the relationship that you build with participants by showing that their stories and company have meaning to you, too.

This discussion aimed to critically evaluate my research process and my success in addressing my research objectives. The exercise revealed how I reproduced some known challenges in participatory research, particularly through the control I held as both researcher and artist. The exercise also suggested how, although needing refinement, illustration and zine-making have value as methods of representation to share stories of disaster. The question, “Whose representation?”, is highly pertinent in studies where researchers work creatively. Addressing the question requires researchers to clarify their ideas of how participant empowerment will be achieved and allows later reflection on whether this was realized in practice. My dual researcher-artist role complicated participant involvement and representation but also offered rich connection with people at Fuego and potential to reach our shared objective of amplifying marginalized voices and communicating disaster to at-risk people, as reflected in this participant interview in March 2024:

*Participant (Morelia)*: Grateful. Very, very deeply for this material that you brought to us. […] But let’s put hope in God that we die in peace and … and ask God for the next generation, that they have – that’s what this material is for, that’s what you are for, that you give this talk, that talk, to those who will come and the young people who are studying. So that they are informed in relation to this, to what we have experienced, because this generation is on its way out, we are already history. Soon we are going to be history. So it is necessary to leave the talk or the knowledge to others. One of these I am going to give to my children. We are going to share it with my children so that they can read it and … it is a story captured in a book that is precisely about this area, right. That’s great. And I thank you very much for this material. It is going to help us a lot, if not us, then the others to come. And that is wonderful.

## Conclusions

This paper describes the process of creating a zine about local people’s experiences of eruptions of Fuego volcano (Guatemala) in the second half of the 20th century. Illustration and zine-making were explored as tools to record older peoples’ experiences of volcanic disaster and to share them with at-risk people in their community. Research involved a series of consultations and participatory workshop activities in 2021–2024. Challenges to participant involvement and representation suggest the need for methodological refinement. However, illustration showed potential in sensitively exploring memories of disaster, while participatory activities allowed people to creatively share stories of Fuego across communities and generations. Critical self-evaluation reveals how my dual researcher-artist role contained tensions that complicated my research objectives but also offered unique advantages to working sensitively with trauma, preserving ageing memories, and enriching existing relationships by “paying attention twice”. These reflections provide insights for other researchers considering arts-based methods as means of representing and amplifying the voices of people affected by disaster.

## Electronic supplementary material

Below is the link to the electronic supplementary material.

Supplementary Material 1

Supplementary Material 2

Supplementary Material 3

Supplementary Material 4

## Data Availability

No datasets were generated or analysed during the current study.

## Footnotes

1 My first volcano zine described the 1902 eruption of Volcán Santa María and the formation of Volcán Santiaguito through the legend of Juan Noj. The zine aimed to communicate volcanological knowledge creatively and was shared at a symposium commemorating the centenary of the 1922 eruption of Volcán Santiaguito.

2 For example, I arranged a consultation with an older man in Panimaché Dos. I expected a 1:1 conversation but I arrived at our meeting to find over 30 people gathered, including the local volunteer disaster reduction group (“COLRED”). My contact had invited other community members to the meeting so that he and his peers could share their experiences of Fuego’s eruptions in the 1960s and 1970s.

3 Morelia was formerly a privately-owned *finca* that produced coffee, a history this participant knew and related to me. I included coffee beans in the drawing to acknowledge this history.

4 Sou developed her comic with low-income families in a Puerto Rican neighbourhood affected by Hurricane María in 2017, while Tatham-Fashanu worked with a diverse group of children aged 4–6 in a primary school in north England.
