# Supplementary material for: Illustrating Fuego: the particular challenges and richness of using arts-based participatory methods to communicate experiences of volcanic disaster
Source: J Appl Volcanol. 2025 Feb 10;14(1):1. doi: 10.1186/s13617-025-00149-0 (PMC11807920; doi:10.1186/s13617-025-00149-0)
Supplement: Supplementary file 1 — Supplementary Material 1 [file 13617_2025_149_MOESM1_ESM.pdf]

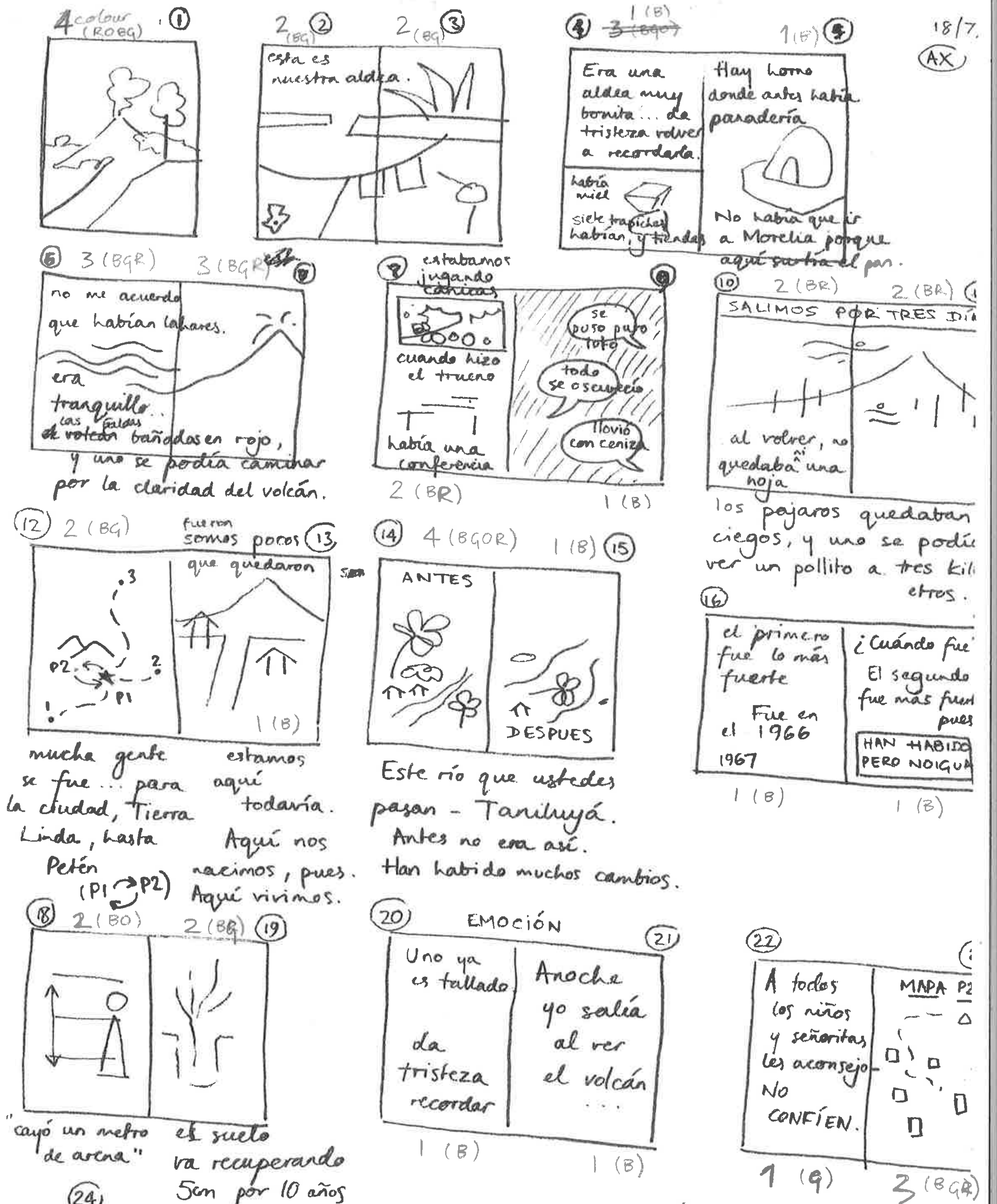

㉔

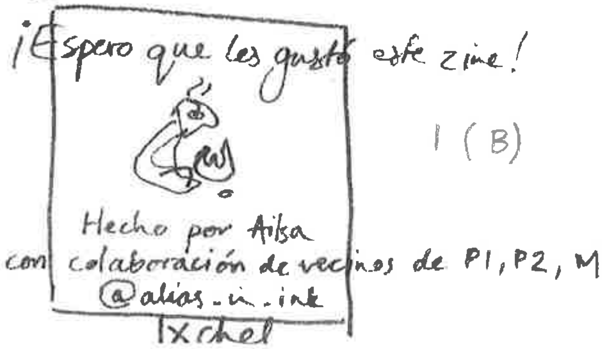

1 (B)

Totals:

colours	pages	
1	~~||||~~ ~~||||~~ |	(11)
2	~~||||~~ |||	(8)
3	|||	(3)
4	||	(2)
		24
