# Supplementary figures and images for: Illustrating Fuego: the particular challenges and richness of using arts-based participatory methods to communicate experiences of volcanic disaster
Source: J Appl Volcanol. 2025 Feb 10;14(1):1. doi: 10.1186/s13617-025-00149-0 (PMC11807920; doi:10.1186/s13617-025-00149-0)

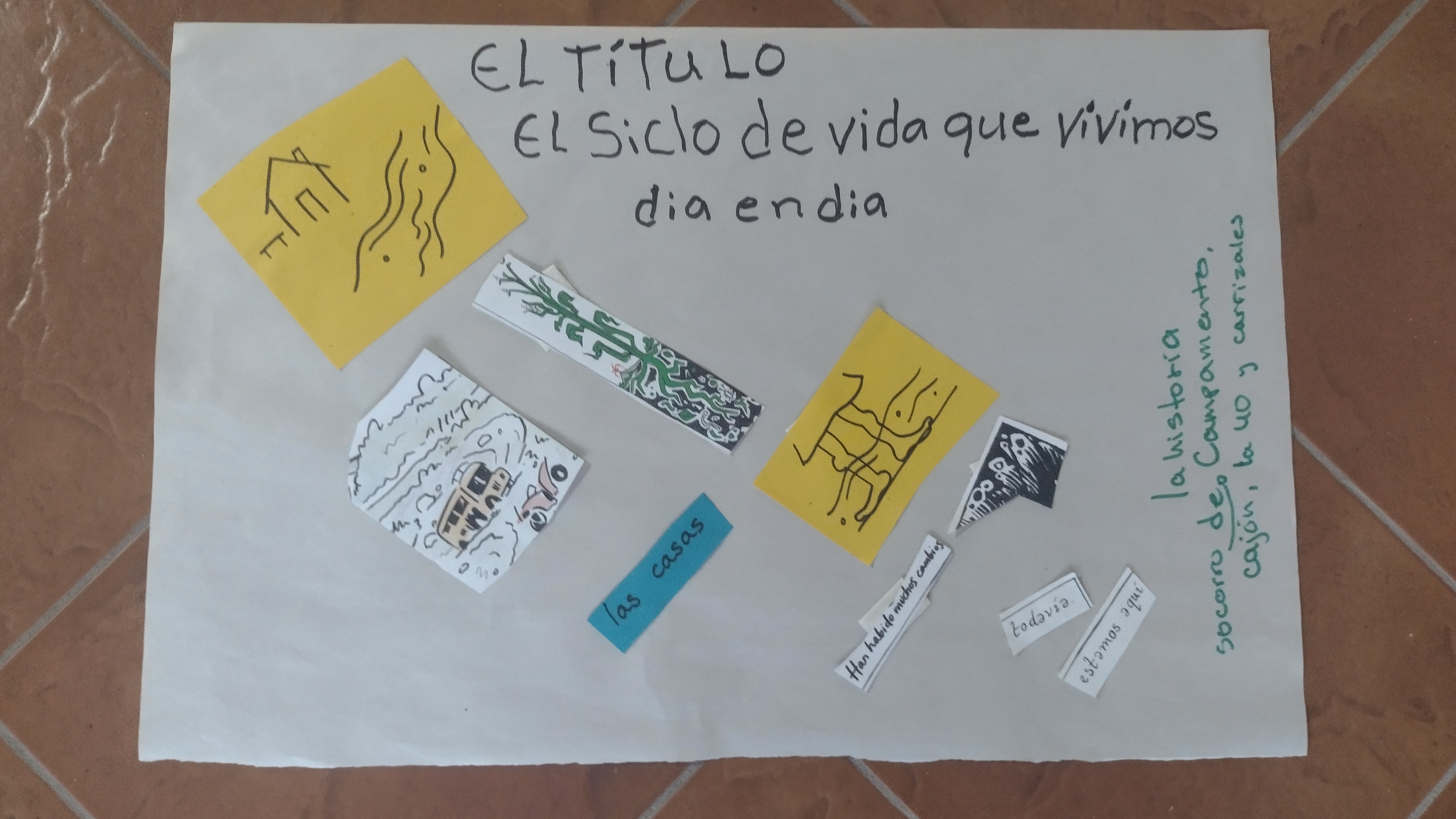

Supplement: Supplementary file 2 — Supplementary Material 2 [file 13617_2025_149_MOESM2_ESM.jpg]

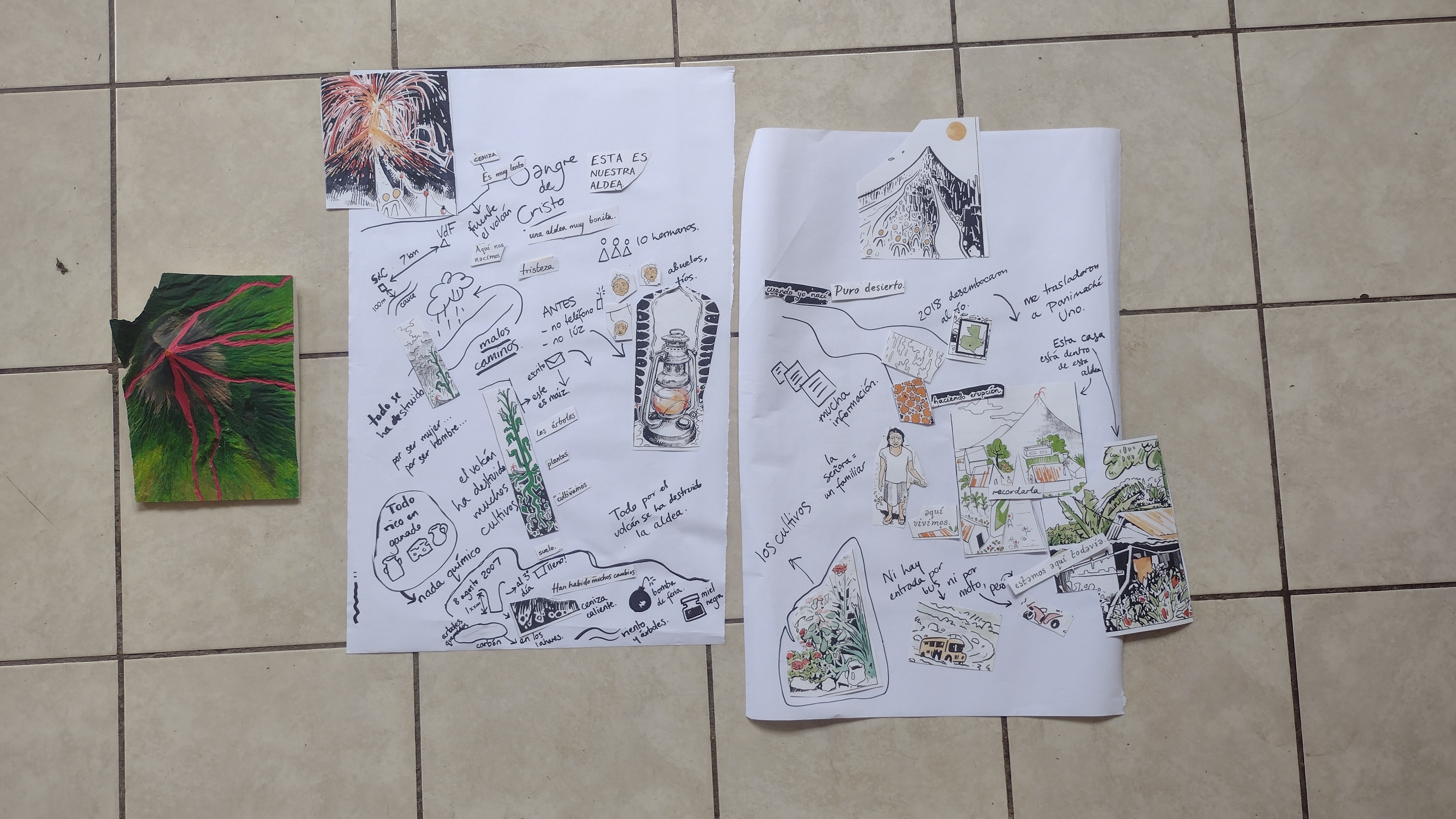

Supplement: Supplementary file 3 — Supplementary Material 3 [file 13617_2025_149_MOESM3_ESM.jpg]
