# Supplementary material for: Illustrating Fuego: the particular challenges and richness of using arts-based participatory methods to communicate experiences of volcanic disaster
Source: J Appl Volcanol. 2025 Feb 10;14(1):1. doi: 10.1186/s13617-025-00149-0 (PMC11807920; doi:10.1186/s13617-025-00149-0)
Supplement: Supplementary file 4 — Supplementary Material 4 [file 13617_2025_149_MOESM4_ESM.docx]

**Ilustrando Fuego: Los desafíos particulares y la riqueza de utilizar métodos participativos basados en las artes para comunicar experiencias de desastres volcánicas**

Ailsa K. Naismith**^1,2^**

***1*** *Escuela de Ciencias de la Tierra, Wills Memorial Building, Universidad de Bristol, Reino Unido*

***2*** *Observatorio de la Tierra de Singapur, Universidad Tecnológica de Nanyang, Singapur*

*Autor correspondiente: ailsa.naismith@ntu.edu.sg*

****NOTA:** La autora principal tradujo este manuscrito del inglés al español utilizando el software DeepL y lo revisó en busca de errores y precisión contextual. Las citas de personas de Fuego aparecen tal y como fueron pronunciadas originalmente en las entrevistas.

**Resumen**

Aunque las catástrofes afectan de forma desproporcionada a las personas vulnerables, sus experiencias a menudo no se cuentan. Los investigadores de desastres utilizan cada vez más métodos de investigación participativa para reconocer y amplificar estas voces ausentes. Los métodos participativos basados en las artes prometen tanto el empoderamiento de la población local como la oportunidad de que los investigadores trabajen de forma creativa y reflexiva. También plantean retos de participación y representación. Este artículo describe el proceso de coproducción de un "fanzine" que describe las experiencias de la población local con las erupciones del volcán de Fuego (Guatemala) y los impactos de estas erupciones que causaron desastres. El fanzine está narrado por la población local e ilustrado por la investigadora (yo misma), que realizó entrevistas semiestructuradas a los residentes para comprender mejor sus experiencias. Se exploran las motivaciones de este proyecto junto con las lecciones aprendidas al llevar a la práctica el diseño de la investigación participativa. Adoptando una perspectiva crítica constructiva sobre mi proceso, documento los retos que encontré en la participación y representación de la comunidad y discuto cómo pueden abordarse en el diseño y ejecución del proyecto. También describo las tensiones y el valor de desempeñar a la vez el papel de investigadora y de artista. Este artículo describe un método abierto de exploración de la investigación en el que se documentan y reflexionan las oportunidades y limitaciones de la representación visual para contribuir a una comprensión compartida y más amplia de las experiencias diversas de desastres volcánicas.

**Palabras clave**

*Fanzines, métodos artísticos, ilustración, investigación participativa, desastre, erupción volcánica*

**Introducción**

La investigación participativa pretende "dar voz a los miembros de grupos marginados, o permitirles que hagan oír su voz" (Bergold y Thomas, 2012, p. 202). Esto es especialmente pertinente en el caso de los desastres relacionados con amenazas naturales, en las que las personas marginadas se ven afectadas de forma desproporcionada, pero a menudo se pasan por alto. Los investigadores de desastres pueden elegir métodos participativos porque, en lugar de tratar a las personas como un grupo homogéneo de "víctimas de desastres", estos métodos capturan sus diversas experiencias (Sou & Hall, 2021) y ofrecen alternativas sensibles al trauma donde los enfoques estándar, como las entrevistas, podrían no ser bienvenidos (Espinoza et al., 2019). Los métodos participativos también prometen a los investigadores una mayor flexibilidad y reflexividad que los diseños de investigación convencionales (Bergold & Thomas (2012), Childs et al. (2017)). Sin embargo, los métodos participativos plantean sus propios retos: trabajar con comunidades que han sufrido desastres requiere tiempo, sensibilidad y un trabajo ético. Quién controla el proceso de investigación es una cuestión compleja (Le De et al., 2014); aunque el control de la comunidad se identifica a menudo como el ideal más elevado de participación, en la práctica rara vez se consigue (Nkombi y Wentik, 2022). La definición de "éxito" cambia con la perspectiva de las partes interesadas (Bergold y Thomas, 2012), y entre los obstáculos prácticos se incluyen las limitaciones de tiempo o energía de los participantes y las concepciones o expectativas de proyecto desalineadas. A pesar de estos innumerables retos, los beneficios de los métodos de investigación participativa perduran. Estos beneficios incluyen una mayor oportunidad para el diálogo, más equidad entre el investigador y los participantes ("respetar y comprender a las personas con y para las que trabajan los investigadores", Cornwall y Jewkes, 1995, p. 1674) y un mayor énfasis en el valor del proceso junto con los resultados.

En las últimas décadas, los métodos participativos se han empleado cada vez más en la investigación sobre catástrofes, ya que tanto las iniciativas mundiales (como el Marco de Sendai para la Reducción del Riesgo de Desastres) como los estudios de casos concretos demuestran su eficacia para comprender los riesgos a los que se enfrentan las comunidades. Sin embargo, sigue siendo difícil garantizar una verdadera participación (Mercer et al., 2008). Además, definir la "verdadera" participación es complicado debido a la gran cantidad de métodos disponibles y a los diferentes niveles de participación comunitaria que implican. Arnstein (1969), estudiando la participación ciudadana en los procesos de planificación en los EE.UU., diseñó una tipología de participación ciudadana con niveles visualizados como peldaños de una escalera. McCall y Peters-Guatin en Wisner et al. (2012) adaptan la escalera de Arnstein a la investigación sobre la reducción del riesgo de desastres (RRD) para categorizar las intensidades de participación que implican los métodos de investigación disciplinarios comunes. Lamentablemente, el peldaño más bajo, *Explotación*, es muy común (Van Niekerk & Annandale, 2013). Los investigadores de catástrofes que deseen trabajar de forma ética pueden elegir métodos con una mayor participación de la comunidad (desde *Intercambio de información* hasta *Transformación,* en la que los miembros de la comunidad se automovilizan e invitan a personas ajenas a la comunidad a colaborar en la investigación). Aunque los métodos descritos en este documento difieren de los descritos por Van Niekerk & Annandale (2013), implican intensidades similares de participación comunitaria y se comparan para evaluar la participación en este estudio (**Figura 6**).

Este documento describe parte de un proyecto de investigación llevado a cabo en 2021 – 2024 con personas de comunidades situadas en las faldas de un volcán activo, el Volcán de Fuego (en adelante "Fuego") en Guatemala. Fuego es un volcán frecuentemente activa de nivel baja. También es capaz de grandes erupciones explosivas (por ejemplo, 10 – 23 de octubre 1974, 3 de junio 2018) cuyos peligros han causado desastres para los miembros de estas comunidades. Las erupciones de la segunda mitad del siglo XX afectaron a los medios de subsistencia agrícolas y contribuyeron a la emigración de las comunidades de los flancos suroccidentales de Fuego (Naismith et al., 2020). Las personas que vivieron estas erupciones recuerdan vívidamente los impactos e incluyen su conocimiento experiencial en sus respuestas a las recientes crisis eruptivas (Naismith et al., en preparación). Sin embargo, muchas de estas personas han sido persistentemente marginadas por el Estado guatemalteco y sus experiencias de erupciones pasadas no han sido contadas**.** Este estudio fue motivado por la prominencia de estas erupciones en la memoria colectiva de estos residentes ahora mayores y el deseo de representar sus experiencias. El estudio pretende explorar el potencial de la ilustración para investigar las experiencias del desastre volcánico. Las preguntas que guían la investigación son: *(¿Cómo) puede utilizarse la ilustración como herramienta para explorar los recuerdos de la catástrofe volcánica?* ¿Y *(cómo) puede utilizarse la ilustración para compartir estos recuerdos con personas en situación de riesgo que no han experimentado tal desastre?* Este proyecto se basa en estudios anteriores que utilizan métodos creativos de representación para profundizar en el diálogo con los participantes:

Cuando los participantes pueden "ver" representaciones de sí mismos en un trabajo creativo visual, están más dispuestos a dar su opinión porque tiene un mayor significado personal, lo que abre espacios de diálogo que pueden llevar al investigador a descubrir ideas más profundas.

Tatham-Fashanu (2023), citado en Sou (2023), p. 328.

El artículo continúa con una revisión de los métodos artísticos en la investigación de catástrofes, introduciendo los fanzines y la ilustración como formas de representación y como posibles herramientas de investigación **(métodos basados en las artes para explorar la respuesta y la recuperación a los desastres)**. **Proceso (métodos y conclusiones)** detalla las fases no lineales del proceso de investigación, incluida la recopilación de historias, la ilustración y la consulta. El **diseño del fanzine** describe las decisiones creativas, mientras que la **discusión** evalúa el diseño y la ejecución del proyecto en relación con la bibliografía existente, debatiendo los retos y los éxitos. Las **conclusiones** resumen los resultados y sugieren futuras mejoras metodológicas.

**Métodos basados en las artes para explorar la respuesta y la recuperación a los desastres**

¿Está la investigación de desastres metodológicamente estancada? Schumann et al. (2019) sugieren que sí, citando la tendencia de los estudios sobre desastres a aferrarse a métodos ortodoxos de recopilación de datos como entrevistas, encuestas y grupos focales. De hecho, están surgiendo muchos métodos innovadores basados en las artes para explorar los desastres, como la fotovoz (Schumann et al., 2019), las entrevistas mejoradas con música (Marsh et al., 2020), los cómics (Sou et al., 2021), la poesía (Miller & Brockie, 2015), las novelas gráficas (Nalla et al., 2022) y la creación de fanzines basados en entrevistas (Valli, 2021). Estos autores dan prioridad a la investigación ética valorando la experiencia individual de los participantes y su aportación al proceso de investigación. Los métodos basados en las artes en la investigación sobre desastres pueden ayudar a las personas a reprocesar el trauma (Huss et al., 2016), diversificar las narrativas de los supervivientes más allá de los relatos homogéneos de "víctimas del desastre" (Sou & Hall, 2023), democratizar el proceso de investigación y permitir a los investigadores trabajar de forma más creativa (Sou et al., 2021). Este cambio de enfoque es paralelo a la defensa de la reducción del riesgo de desastres basada en la comunidad (CBDRR, por sus siglas en inglés), ya que los investigadores abogan por un cambio de los enfoques de RRD paternalistas y verticalistas a aquellos que requieren que las autoridades se comprometan directamente con las personas en riesgo. Stone et al. (2014) examinan la red *vigía* del volcán Tungurahua (Ecuador) para mostrar cómo la colaboración entre científicos y comunidades puede mejorar la mitigación del riesgo, la vigilancia de las amenazas y la capacidad de las comunidades. Paton et al. (2022) identifican la necesidad de incluir los procesos sociales y culturales en el desarrollo de la CBDRR. A través de un estudio de caso, los autores vislumbran cómo las canciones y el arte -como representaciones de contextos socioambientales – podrían utilizarse para desarrollar creencias y acciones de RRD, aunque sugieren que las artes visuales pueden enfrentar mayores desafíos de accesibilidad que la música (Paton et al., 2022).

Los fanzines (pronunciado como "*maga-zine*") son publicaciones amateurs de pequeña tirada creadas por aficionados a un tema para conectar con otros devotos (Triggs, 2006). Los fanzines tienen su origen en el mundo de la ciencia ficción de los años treinta, cuando los aficionados creaban y autoeditaban revistas para rellenar los huecos entre las publicaciones oficiales (Coppa, 2006). Dado que su formato permite a los creadores "crear tu propia cultura y dejar de consumir la que está hecha para ti" (Duncombe, 1997, p. 2), los fanzines han sido un medio de expresión favorito entre varias comunidades contraculturales del siglo XX, como la escena queer neoyorquina, los punks de los setenta y las Riot Girrrls (Triggs, 2006; Romano, 2023). Los fanzines han perdurado como forma de comunicación más allá de sus orígenes anteriores al Internet (Romano, 2023). El internet ha permitido que los fanzines y sus comunidades prosperen tanto en formato digital como físico. Tal vez sea porque su atractivo original se mantiene: conectar a un pequeño número de personas con ideas afines a través del intercambio de información y opiniones en un estilo visual idiosincrásico. Los creadores de fanzines comparten la motivación de autopublicarse con los artistas de vanguardia del siglo XX: "el deseo de compartir ideas, ideología, arte, etc., ... que de otro modo estarían infrarrepresentados" (Gardner, 2023, p. 4); por tanto, como artefactos tangibles producidos por y para comunidades marginadas, los fanzines tienen importancia tanto académica como social. Los fanzines también proporcionan una forma alternativa de hacer memoria en respuesta al trauma (Cooper, 2024). Por su accesibilidad y facilidad de distribución, los fanzines ofrecen una interesante posibilidad de utilizar las artes visuales para explorar experiencias de desastres y facilitar el desarrollo de creencias y acciones de RRD.

Para compartir su propia posición con respecto a los fanzines y su cultura, los estudiosos del fanzine suelen narrar su conexión personal con la creación de fanzines (Legendre, 2023). He sido un artista durante muchos años, pero descubrí fanzines más recientemente a través de la cultura local en Bristol. Hice fanzines a lo largo de 2020 en los encierros de la pandemia COVID. Hacer fanzines dio forma a este tiempo liminal (Cooper, 2024). Además, su materialidad adquirió "una nueva dimensión afectiva durante el cierre" (Cooper, 2024): como la vida se trasladó repentinamente a Internet, su tangibilidad se hizo atractiva. Esta respuesta se hace eco de la perspectiva de Piepmeier de los fanzines como "comunidades encarnadas": "En un mundo en el que cada vez somos más los que pasamos todo el día frente al ordenador, los fanzines nos reconectan con nuestros cuerpos y con otros seres humanos" (Piepmeier, 2009, p. 58). Los fanzines me permitieron volver a conectar con el exterior y con otras personas mientras el encierro inhibía los viajes y las conexiones en persona (p. ej., un fanzine de una "búsqueda del tesoro" en mi barrio publicado más tarde en una revista comunitaria (Up Our Street, 2021); un mapa de fantasía de mi doctorado, publicado en el blog de la Universidad de Bristol para estudiantes de doctorado (BDC, 2020)). Creé fanzines para explorar de forma creativa la historia y mitología eruptiva de los volcanes^[[1]](#footnote-1)^ . Estas experiencias me motivaron personalmente para explorar las experiencias de la población local con el volcán de Fuego a través de la creación de fanzines y la ilustración.

Los métodos de este documento y de los estudios citados requieren un tiempo y un esfuerzo considerable. Algunos investigadores consideran estos métodos una forma de resistencia contra las exigencias del mundo académico moderno, incluidos los defensores de la "escolaridad lenta", un movimiento feminista que sostiene que la escolaridad de calidad debe tener tiempo para madurar sin prisas (Mountz et al., 2015). Los estudios que exploran los desastres a través de métodos basados en las artes suelen requerir que los investigadores generen confianza con las personas en riesgo a lo largo del tiempo (p. ej., un año en Puerto Rico antes y después del huracán María (Sou, 2023); tres años en tres estados de la India afectados por inundaciones (Nalla et al., 2022)). He trabajado con personas de comunidades cercanas a Fuego durante siete años (2017 – 2024). He desarrollado relaciones de confianza con muchas personas, lo que motivó nuestro deseo compartido de representar sus historias con sus propias palabras y mis ilustraciones.

**Proceso (métodos y resultados)**

**La figura 1** es una crónica de las etapas clave de este estudio**.** Aunque el fanzine incluye algunas palabras de entrevistas de 2019, la cronología formal comienza en noviembre de 2021 con el primer periodo de trabajo de campo centrado exclusivamente en las experiencias de la gente con las erupciones de Fuego en la segunda mitad del siglo XX.

**Figura 1:** cronograma visual del proceso de investigación.

El fanzine cuenta la historia de los impactos de las erupciones de Fuego en la segunda mitad del siglo XX a través de las voces de la población local. Estos eventos también se describen a través del análisis de entrevistas en Naismith et al. (en preparación). En entrevistas y conversaciones en 2019 – 2024, las personas de tercera edad compartieron cómo estas erupciones afectaron negativamente a sus vidas y a los resultados de sus medios de subsistencia. Se sintieron continuamente marginados por el gobierno guatemalteco, a quien acusaron de no abordar los impactos persistentes de la actividad de Fuego (p. ej., la construcción de puentes para que las personas crucen de manera segura los ríos por donde descienden los flujos volcánicos). Expresaron su deseo de registrar permanentemente sus experiencias y compartirlas con los más jóvenes de sus comunidades, que no han vivido una serie de grandes erupciones comparables a las de la segunda mitad del siglo XX. Debatimos sobre las formas de registrar y compartir estos testimonios. Mientras que algunas visitas a la comunidad se convirtieron orgánicamente en sesiones de narración de historias^[[2]](#footnote-2)^ , los residentes mayores también querían un documento tangible para llevar sus voces tanto dentro como fuera de su comunidad. Varias personas sugirieron la publicación de un libro, pero esta propuesta planteaba el reto de los distintos niveles de alfabetización y la escasez de tiempo y recursos. Yo propuse un fanzine ilustrado como alternativa accesible – fácil de reproducir y difundir, barato, visualmente atractivo y sin mucho texto – que se ajustaba a sus intenciones y al entorno de Fuego. También es una forma de arte que yo practico y disfruto (véase **Métodos basados en el arte para explorar la respuesta y la recuperación tras un desastre).** Cuando propuse un fanzine, describiéndolo como un "libro sencillo con dibujos" en el que podríamos documentar sus historias a través de conversaciones e ilustraciones, la gente aceptó la idea.

Aunque el arte en la investigación de catástrofes no es algo nuevo (véase **Métodos basados en el arte para explorar la respuesta y la recuperación tras un desastre**), este proyecto fue inusual en el sentido de que asumí los papeles de investigadora y artista. En teoría, se podría hacer un número ilimitado de ilustraciones sobre Fuego; en realidad, el tiempo era limitado tanto para mí como para los participantes. Por lo tanto, tuvimos que priorizar qué historias documentar. Hice los primeros bocetos para este proyecto en 2021 – 2022, ilustrando recuerdos de entrevistas con personas mayores que viven en comunidades en los flancos suroeste de Fuego, principalmente las comunidades de Morelia, Panimaché Dos y Panimaché Uno (Naismith et al., en prep). Dibujé para personas con las que podría organizar otra reunión para asegurar la oportunidad de retroalimentación (como las consultas descritas por Tatham-Fashanu (2023) y Sou (2023)). Por ejemplo, un boceto de una planta de café que lucha por crecer en la tefra depositada por las erupciones de los años sesenta y setenta ilustró las experiencias de un agricultor que vive cerca de Morelia (**Figura 2**). Aunque estos intercambios fueron en gran medida positivos (págs. 11 y 12), el tiempo que requirieron, unido a las exigencias de otras investigaciones, me impidió organizar consultas en grupo con la gente sobre lo que debía registrarse en el fanzine. Estas limitaciones y mi doble papel de investigadora-artista limitaron la participación de la comunidad en el desarrollo del fanzine, dándome un control significativo sobre los aspectos tanto creativos como de investigación (ver **Discusión**).


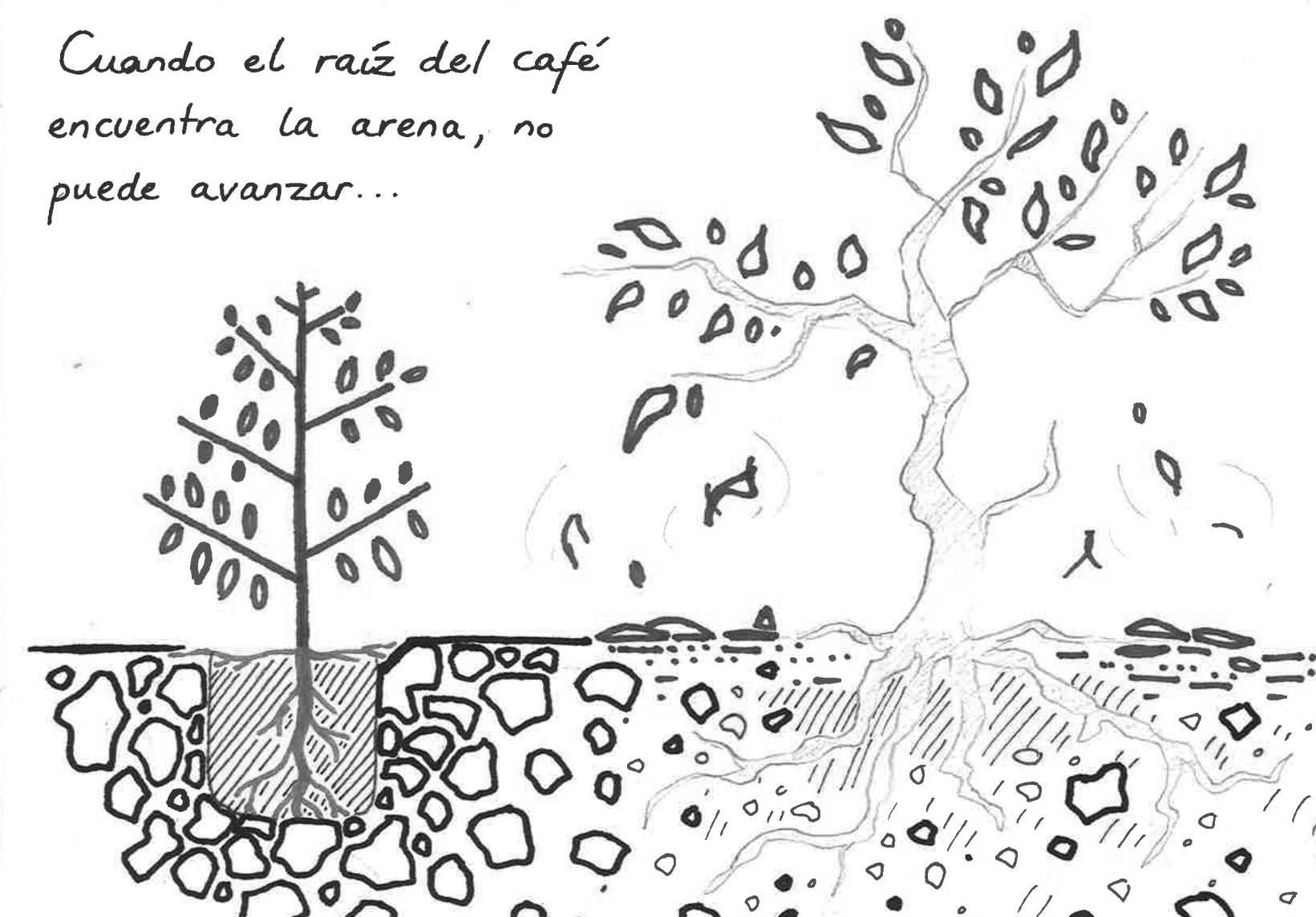


**Figura 2:** un boceto que hice para un participante para ilustrar sus experiencias sobre los efectos de la tefra volcánica en las plantas de café.

En las pocas consultas que realicé a los participantes a principios de 2022, los dibujos que había hecho de sus historias recibieron una respuesta muy positiva. Cuando se les presentó el boceto, las personas expresaron una serie de emociones al ver su experiencia representada en un dibujo. A continuación, hablaron de su recuerdo con mayor profundidad. Este es el intercambio que tuve con el participante cuyas experiencias inspiraron el boceto en la **figura 2**:

Participante (Morelia): Y qué bien señor, que usted haya elaborado esto, porque si se ve que lo que hemos explicado lo han captado cabal, va, porque así es nuestra realidad. Tenemos que hacer un hoyo. Y llenarlo con broza, con materia orgánica que botan otros árboles para llenar el hoyo aquí. Metemos la mata y crece y ahí abonamos, ahí regamos. Y ahí cosechamos. Pero una vez que ya la mata consumió. Lo que hay aquí, hay que reponerle esa …

Un anciano de Morelia recuerda un langostino que le cayó del cielo en las manos durante una erupción apocalíptica (**SM-2,** p. 10). Escuché múltiples versiones de este incidente, con algunas personas relatando un pez en su lugar. Todos los narradores relacionaron el incidente con una gran erupción productora de tefra y me preguntaron si implicaba que el volcán estaba conectado con el mar. La aparición recurrente del langostino en la memoria local lo implicaba como un elemento importante en las historias que la gente contaba sobre Fuego, y dio otra oportunidad para explorar cómo la ilustración podía profundizar en el diálogo sobre estas erupciones. Cuando le entregaron el dibujo, el hombre expresó su alegría y se explayó sobre su recuerdo:

Participante (Morelia): Si, pues. Aquí están otras casitas, ve. Y tantas cositas. … Y mire, todo esto. Aquellos otra casa. Hicieron bien hecho ustedes [senalando el dibujo].

Investigadora: ¿Ah, sí, le gusta? Eso yo lo puse como … Este es Morelia yo puse, porque mira, se pueden mirar las semillas de café^[[3]](#footnote-3)^. Entonces yo dibujé Morelia, y el camino por Panimaché Dos.

Participante: Sí, sí, sí, sí. Este es el camino.

Investigadora: Eso, eso. Entonces justamente subiendo en camino de Morelia para Panimaché Dos.

Participante: Para Panimaché Dos es este. Antes. Antes este era Panimaché Uno. Más antes este era Panimaché Uno. Pero como toda la gente por el volcán se fueron huyendo.

A pesar de mi extenso trabajo previo en estas comunidades, estas consultas tuvieron una riqueza única. La respuesta de la gente al ver sus experiencias representadas mediante ilustraciones reflejaba las conclusiones de Tatham-Fashanu (2023) (citada en la pág. 5) y me conmovió de manera inexpresable. Crear ilustraciones para la gente de Fuego, y compartirlas con ellos, parecía "ofrecer... un testimonio directo sobre el mundo que rodeaba a otras personas en otros momentos... de [una] manera... más precisa y rica que la literatura" (Berger, 1972, p. 1). Reflexionando sobre el proyecto tras su finalización, he intentado articular esta cualidad (véase **Discusión,** págs. 27 – 28).

Mi siguiente oportunidad para realizar trabajo de campo fue en noviembre de 2022. Dado que el trabajo de campo anterior había demostrado la gran cantidad de tiempo que se necesita tanto para la ilustración como para la consulta, pero no podía consultar directamente a las personas en el verano de 2022, desarrollé un método alternativo. Ilustré temas y frases clave de las entrevistas con personas mayores; la recurrencia de los temas indicaba su importancia en la memoria colectiva. El conjunto de datos comprendía 45 entrevistas con 57 personas en dos períodos de estudio (feb – abr de 2019, nov de 2021 – abr de 2022); los detalles completos aparecen en Naismith et al. (en preparación)). Las entrevistas siguieron procedimientos éticos, incluida la obtención del consentimiento de los participantes, la garantía de confidencialidad y el intercambio de datos de contacto con los participantes. Este estudio se realizó íntegramente en español; todas las citas de los residentes de Fuego han sido traducidas al inglés por mí específicamente para la versión inglés de este artículo. Para identificar las frases clave, realicé consultas de texto en NVivo12. Los resultados de las consultas fueron: "*todo se oscureció*" (refiriéndose al cielo oscurecido por la ceniza) y *"después vinieron los lahares*" (recordando los flujos de lodo que descendían por los barrancos de Fuego tras las grandes erupciones). Los temas principales se identificaron mediante sesiones iterativas de codificación, siguiendo un enfoque flexible descrito por Deterding & Waters (2021). Estos temas incluyeron la migración alrededor de Fuego y los eventos sociales que coincidieron con las erupciones (p. ej., Fuego interrumpiendo la inauguración de una iglesia). Aunque algunas historias de erupciones contenían elementos fantásticos (p. ej., la caída del langostino), la gente generalmente no contaba o conocía leyendas sobre Fuego. A través de este proceso de codificación, empezaron a surgir frases y temas clave que eran colectivamente significativos y ricos en sentidos, y por tanto adecuados para la ilustración. En julio de 2022 realicé un guión gráfico para el fanzine (véase **SM-1**) y solicité financiación inicial a una beca de un consejo nacional de las artes. Me propuse utilizar las ilustraciones en consultas con los participantes cuyos recuerdos se ilustraban para pedir sugerencias y dirigir el desarrollo del fanzine en un proceso iterativo y colaborativo (como en Sou & Hall, 2023). Imprimí las ilustraciones en forma de folleto en octubre de 2022. Este formato permitía compartir las ilustraciones en una o varias páginas.

El trabajo de campo de noviembre de 2022 incluyó varias consultas individuales y en grupo y dos talleres participativos. Invité a alguien de la comunidad local para facilitar por su conocimiento del contexto cultural local y su presencia familiar que daría confianza a la gente para compartir. Un facilitador de Panimaché Uno ayudó en algunas consultas, y otro de Panimaché Dos facilitó el taller de esa comunidad. Las consultas tuvieron lugar en Morelia, Panimaché Dos y Panimaché Uno. Concerté las consultas por teléfono o en persona. La mayoría de las personas se mostraron satisfechas de ser contactadas y aceptaron participar. Sin embargo, los participantes de noviembre tuvieron opiniones más dispares que los de marzo. Durante una consulta de grupo en Panimaché Uno, tres hombres que habían sugerido documentar sus experiencias en un libro miraron brevemente mis ilustraciones de sus recuerdos antes de volver a compartir sus historias. Tuve varias interacciones similares durante este trabajo de campo. Algunas consultas produjeron un diálogo más profundo. Una mujer de Panimaché Dos encontró al principio poco que revisar en mi ilustración de su memoria:

Participante (Panimaché Dos): No sé qué usted quiere. Más, más información.

Compañera de investigadora: Más información.

Investigadora: O como, por ejemplo. Ese, de que estábamos hablando. Está … ¿Queda bien, o falta algo? Porque me imagino … esto es solo de mi imaginación, verdad. Entonces puede ser que hay cosas que yo he hecho, que tal vez no aparecen bien. Como este, “Llovió de ceniza”, ¿verdad? Y no era ceniza, sino que era arena.

Hija de participante: Quiere que le ayude, que todo esté bien.

Participante: Ah, ir corrigiendo.

Investigadora: Mm-hm. Porque seguro es que han errores, verdad. Esto salió –

Participante: No, casi – casi todo está bien. Mm-hm.

Sin embargo, más tarde se detuvo en mi ilustración de otro de sus recuerdos, una erupción que interrumpió la inauguración de una iglesia (**SM-2,** p. 8). Trazando la ilustración con el dedo, explicó:

Participante (Panimaché Dos): [mirando la página] las ollas de comida están.

Investigadora: Ah, sí. ¿Qué están, estaban cocinando?

Participante: Cuando fue lo de la arena, fue caldo de res. […] Yo tenía nueve años.

Hija: Ya tiene sesenta y siete.

Participante: Y ahorita tengo sesenta y ocho. […] Yo tenía nueve años cuando fue eso. Me acuerdo yo, que nosotros, con mi primo [fueron allí]. Por eso se huía como que era de [pacaya] granito, pero ¡que arenizo grande! Y todos estaban en la carpa. Los hermanos con barras así, porque como … se sabe que la carpa, va. La lona. Se hundía.

Las consultas en las que compartí una o dos ilustraciones fueron las que provocaron más diálogo. Los participantes utilizaron la ilustración para hablar de su recuerdo y relacionarlo con otros, y cuando les pedí opiniones sobre la ilustración, hicieron comentarios o sugerencias que anoté en la página (**Figura 3).** Tras las consultas, registré mis observaciones y reflexiones mediante notas de campo y grabaciones de audio.


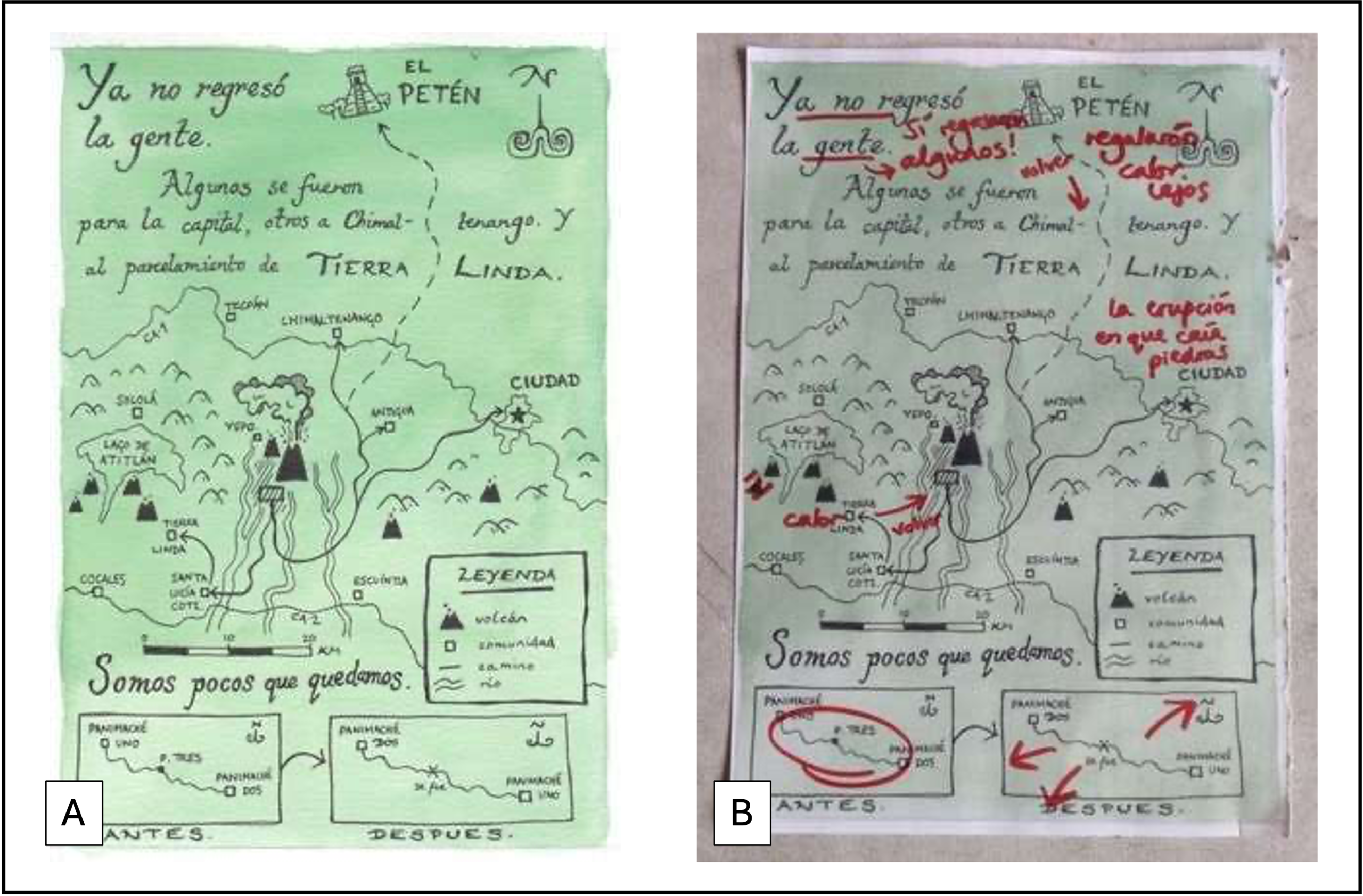


**Figura 3:** ilustraciones de ejemplo del trabajo de campo en noviembre de 2022 que muestran (**a**) una sola ilustración del fanzine; (**b**) la misma ilustración anotada durante una consulta con participantes en Panimaché Uno. Había hablado previamente con estas personas en una entrevista en la que recordaron la migración de Fuego (el tema de esta página).

Realicé dos actividades de talleres participativos en noviembre de 2022. La primera fue dentro de un taller más amplio dentro del proyecto *Ixchel* para mujeres alrededor de Fuego organizado por las Dras. Teresa Armijos Burneo y Cristina Sala Valdez en el pueblo de Siquinalá. El segundo fue con un grupo de mujeres líderes en Panimaché Dos. La actividad del taller constó de tres etapas. Primero, abría el espacio presentándome e invitando a todos a hacer lo mismo, y explicaba que el propósito de esta actividad era reunirnos para intercambiar experiencias de vivir junto al Fuego. En segundo lugar, repartí una hoja de papel grande con la siguiente pregunta: "*Háblame de tu comunidad y de su historia con Fuego*". A los participantes se les ofrecía papel, bolígrafos y recortes de ilustraciones y palabras del fanzine, se les daba tiempo para crear sus historias y luego compartirlas con los demás. Por último, el grupo se reunía para debatir lo que habían aprendido. En la práctica, la actividad funcionó de forma diferente cada vez. En Panimaché Dos, las mujeres trabajaron juntas en una sola hoja que incluía sus propias experiencias y los relatos de los ancianos sobre el pasado de la comunidad (**Figura 4a**). En Siquinalá, las mujeres de distintas comunidades se turnaron para contar sus historias individuales, utilizando palabras e ilustraciones del folleto como apoyo (**Figura 4b**). Aunque los talleres eran muy interactivos, los niveles de participación variaban de una persona a otra. Desarrollé el diseño de la actividad durante mi estadía en el campo, en parte debido a mi preocupación por el nivel de implicación de la comunidad en el proceso de elaboración del fanzine (véase **la Discusión**)**.**


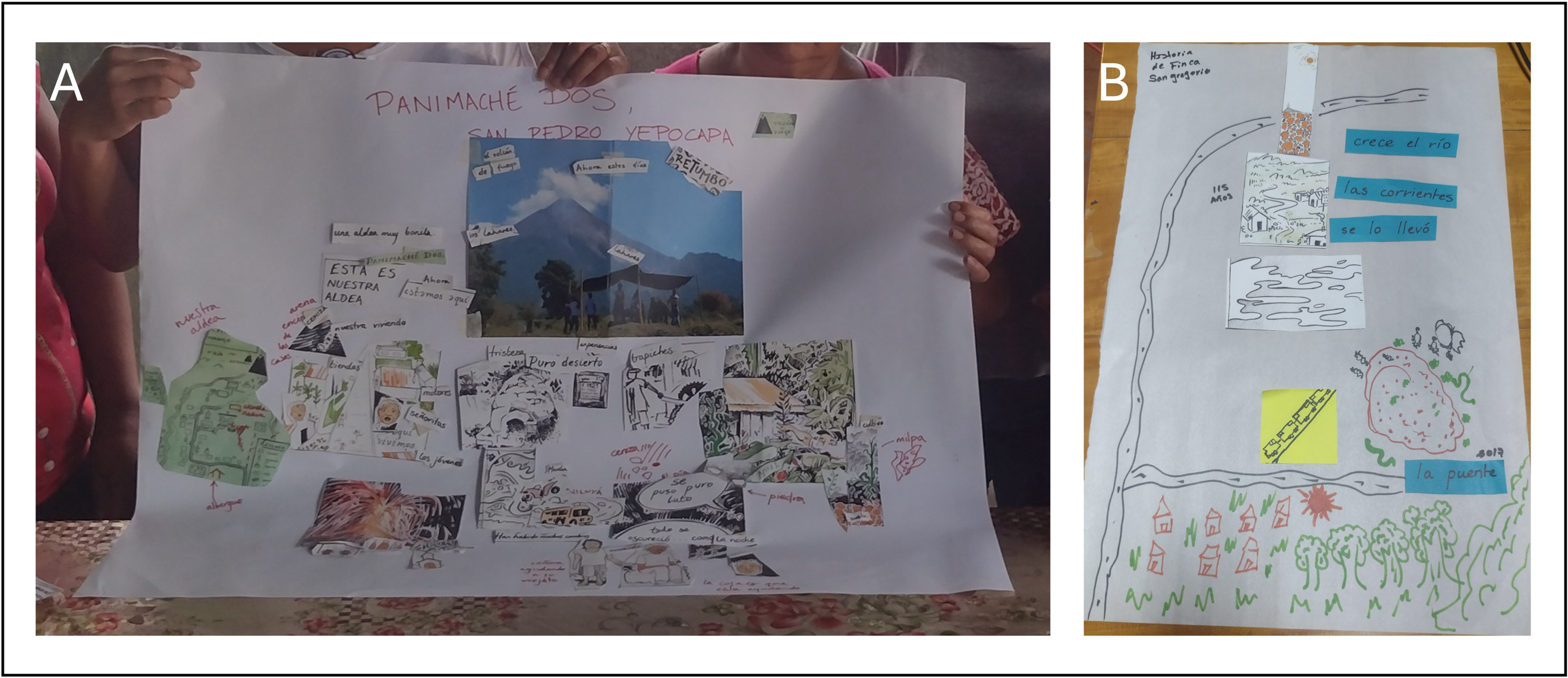


**Figura 4**: resultados de las actividades participativas realizados en noviembre de 2022 con (**a**) mujeres de Panimaché Dos y (**b**) mujeres de Siquinalá. La obra en (**a**) fue desarrollado colectivamente por las participantes, mientras que (**b**) fue creado individualmente por una participante de la Finca San Gregorio (una comunidad en la bocacosta al sur de Fuego regularmente afectada por inundaciones crecidas por sedimentos de la actividad volcánica) y compartido con el grupo.

Durante mi siguiente visita al campo, en marzo de 2023, me propuse compartir una segunda edición del fanzine, que incorporaba los comentarios de los participantes a través de ilustraciones revisadas y nuevas que había realizado desde noviembre. Sin embargo, esta visita resultó ser la más complicada entre todas mis visitas al campo por este estudio. Muchos participantes no estaban disponibles para reunirse conmigo durante mi breve visita (una semana), ya que coincidía con la temporada alta de la zafra (la cosecha de caña de azúcar), cuando muchos hombres mayores estaban trabajando en los campos. Para aprovechar bien el tiempo y explorar mi segunda pregunta de investigación, quedé con algunas personas de Panimaché Dos que aceptaron evaluar el fanzine. Aunque estas personas no habían participado en las consultas anteriores, representaban a personas en situación de riesgo que los residentes mayores habían identificado anteriormente como parte del público al que iban dirigidas sus historias documentadas. Una mujer que había vivido en Panimaché Dos durante décadas, y con cuyo marido había hablado extensamente sobre Fuego, dijo: *"Esta historia no es de nosotros*". Este sorprendente comentario me animó a reflexionar críticamente sobre las limitaciones y las ventajas de hacer fanzines para compartir recuerdos con personas en situación de riesgo que no han vivido un desastre volcánico. Estas reflexiones aparecen en **Discusión** (págs. 24 – 25).

Tuve una última oportunidad de evaluar el fanzine en marzo de 2024 en una visita a Panimaché Uno. Invité a miembros de la comunidad a una reunión en el observatorio de Fuego. Aunque algunos admiraron brevemente el folleto y luego volvieron a compartir otras historias, una mujer comentó:

Participante (Panimaché Uno): Lo que está en los libros … para mí, pues, me parece bien. Me gusta. Yo crecí en otro Panimaché. Dos. Había también panaderías, había otras cosas. Muy bonito. Y viendo estos libros, recuerda uno lo que los abuelos contaron […] Y lo que es de esta aldea, pues, no tenía conocimiento de nada. Y viendo esto libro, uno ya … ve uno lo que ha ido antes. Y todo se destruyó. Porque ahora, ya no se mira nada de esto. Panadería y eso, ya no hay nada.

**En la figura 5** se indica el tiempo que han requerido los elementos (consultas y talleres participativos) que componen mi proceso de investigación. "COMPLETO" representa un punto de pausa adecuado para una línea de investigación más que un final. La siguiente sección, **Diseño del fanzine,** explora brevemente las decisiones implicadas en la elaboración del fanzine. **La discusión** evalúa críticamente este enfoque y los retos que encontré, y ofrece orientación a otros investigadores que consideren la ilustración o la creación de fanzines como formas de representación para explorar la experiencia vivida
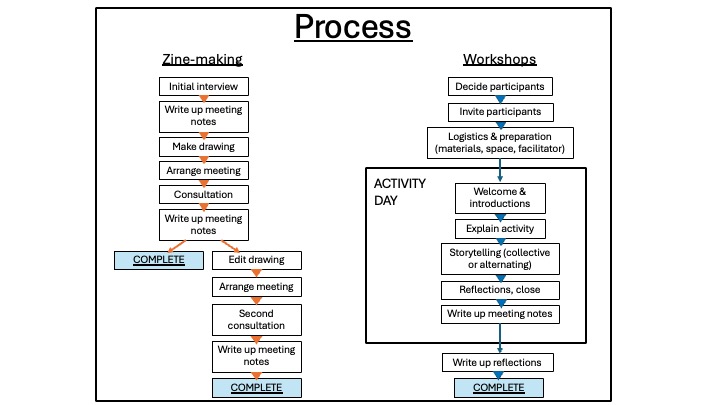
de los desastres.

**Figura 5**: diagrama de los procesos de investigación que seguí en este proyecto, en el que se describen tanto las consultas sobre ilustraciones como las actividades participativas en grupo. Ambos procesos implican una inversión grande de recursos. Los retos y éxitos de llevar este diseño a la práctica aparecen en la **Discusión**.

**Diseño del fanzine**

El fanzine se montó a partir de bocetos, dibujos e ilustraciones que realicé en 2021 – 2023 (véase **Proceso (Métodos y resultados))**. Algunas piezas se incluyeron sin revisar en la versión final, mientras que otras (por ejemplo, las páginas 8 y 29) se revisaron tras realizar consultas. Dada mi estrecha relación con la gente de Fuego, nuestro deseo compartido de contar sus historias sobre las erupciones que causaron desastres y mi experiencia en la elaboración de fanzines, quería crear una obra visualmente atractiva en la que las ilustraciones acompañaran a citas directas de la experiencia vivida por la gente para compartir sus voces sin filtros. Las historias que la gente había compartido conmigo a lo largo de los años en Fuego me ofrecían la posibilidad de crear una obra que fusionara la historia eruptiva y la mitología y proporcionara una forma alternativa de hacer memoria en respuesta al trauma (véase **Métodos basados en las artes para explorar la respuesta y la recuperación tras un desastre**). Aunque al principio me planteé hacer un fanzine con un sencillo formato desplegable, la gente había compartido tantas historias conmigo que este formato me pareció limitante, incluso después del proceso de selección de citas y priorización de historias (pág. 13). La versión final del fanzine tiene 34 páginas. Estas páginas contienen una fracción de las historias en torno a Fuego, y sin embargo requirieron un enorme esfuerzo y tiempo. El objetivo del fanzine era reconocer la riqueza y viveza de las historias de las personas mayores y su generosidad a la hora de compartirlas, así como una forma de capturar un entorno y las vidas en él marginadas y tan raramente vistas. Las opciones de diseño del fanzine se basaron en gran medida en mis experiencias, y en la siguiente sección se analizan la ética y las tensiones de este enfoque.

**Discusión**

Este estudio se propuso explorar dos cuestiones: *(¿Cómo) puede utilizarse la ilustración como herramienta para explorar los recuerdos del desastre volcánico?* Y *(¿cómo) puede utilizarse la ilustración para compartir estos recuerdos con personas en situación de riesgo que no han experimentado tal desastre?* Basándome en la bibliografía existente, evalúo críticamente hasta qué punto mi investigación ha respondido a estas preguntas. La evaluación revela retos y éxitos en dos temas clave: participación y representación.

A lo largo de este estudio, me pregunté hasta qué punto mi proceso era realmente participativo. La flexibilidad de la investigación participativa complica la evaluación de esta cuestión, ya que la implicación de la comunidad varía incluso dentro de un mismo método. Decidí evaluar la participación de la comunidad en mi proceso comparándola con los marcos existentes. La escala de participación ciudadana de Arnstein (1969) se ha adaptado a la investigación de catástrofes para visualizar las diferentes "intensidades" de participación (Van Niekerk & Annandale (2013, pág. 163)); he modificado esa figura para mostrar los métodos de mi proceso y mi evaluación de la intensidad de participación de cada método (**Figura 6).** En la intensidad más baja, el *Intercambio de información* implicaba presentar varias páginas o un fanzine entero a la gente e invitarles a dar su opinión durante las visitas al campo (p. ej., consulta grupal, pág. 14; recuadro naranja, **Figura 6**). La *consulta sobre temas y cuestiones* implicaba compartir una ilustración con las personas para suscitar un diálogo más profundo sobre sus recuerdos (p. ej., consultas, págs. 11 y 12; recuadro verde, **Figura 6**). Las actividades del taller de noviembre de 2022 oscilaron entre la *Consulta sobre resultados y conclusiones provisionales* y la *Colaboración*: los participantes trabajaron de forma interactiva y decidieron la dirección de la investigación (págs. 16 – 17; recuadro azul, **Figura 6**). En el taller de Panimaché Dos, el grupo trabajó en conjunto para crear una historia colectiva de la comunidad que incluyera tanto sus experiencias vividas como las historias escuchadas de los ancianos. En el taller de Siquinalá, las mujeres se alternaron como narradoras y audiencia, y las narradoras utilizaron materiales de fanzines para contar sus propias experiencias sobre Fuego. Estas actividades son prometedoras para comunicar el desastre volcánico a personas en riesgo sin experiencias vividas similares, compartiendo historias entre comunidades (Siquinalá) o entre generaciones (Panimaché Dos). El uso de ilustraciones por parte de los participantes demuestra cómo las formas culturales (arte, historias, rituales) ayudan a las personas a expresar sus experiencias dentro del contexto y la comprensión de su comunidad (Huss et al., 2016).

##
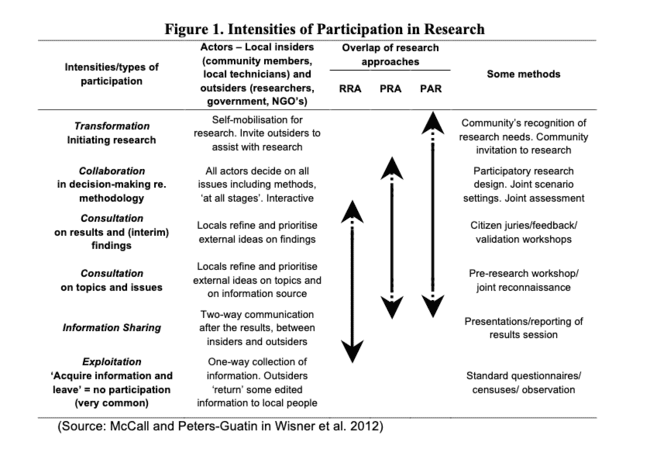


**Figura 6**: intensidades de participación en la investigación de desastres (de Wisner et al. (2012) en Van Niekerk & Annandale (2013, p. 163). Los recuadros de color (añadidos por mí) indican las intensidades de participación a las que invitan mis métodos.

Examinar los procesos de consulta ofrece otra forma de evaluar la participación de la comunidad, ya que suelen detallarse en la investigación participativa. Sou (2023) describe su proceso de consulta para *After María:* "Invité a los participantes a dar su opinión... La gente se sintió cómoda dirigiendo, criticando y sugiriendo enmiendas e ideas porque podían ver literalmente sus entornos" (Sou, 2023, pp. 327 – 328). Del mismo modo, Tatham-Fashanu (2023) describe la co-creación activa de cómics con los participantes. En mis consultas, los comentarios de los participantes eran más equívocos: algunos hacían sugerencias con facilidad, mientras que otros se limitaban a dar su aprobación; rara vez criticaban el trabajo. ¿A qué se deben estas diferencias? La falta de familiaridad cultural no parece significativa: ni Tatham-Fashanu ni Sou trabajaron con participantes que estuvieran profundamente familiarizados con los cómics^[[4]](#footnote-4)^ , y ambos sostienen que los métodos visuales son universalmente accesibles. Sin embargo, la calidad del arte presentado para consulta puede haber contribuido a estas diferencias. Los cómics de Tatham-Fashanu incluyen figuras de palo, que ella describe como un lenguaje visual sencillo y comprensible; Sou compartió bocetos a lápiz. Mis ilustraciones más detalladas (**Figura 7)** pueden haber limitado los comentarios de los participantes porque estaban satisfechos con la ilustración existente, porque nuestra relación personal significaba que se sentían incómodos criticando mis dibujos, o porque no estaban motivados para participar porque no veían la oportunidad de "influir profundamente" la investigación (Sou et al., 2021). El examen de mis métodos reveló tensiones entre mis papeles de investigadora y artista, ya que la creación de fanzines y la ilustración invitaban a niveles de participación diferentes de los inicialmente previstos. Estas tensiones complicaron mis objetivos de investigación.

La creación de fanzines puede lograr una gran implicación de la comunidad cuando los investigadores invitan a los participantes a dirigir el proceso (Valli, 2021). Incluso los fanzines creados en solitario pueden invitar a la participación de la comunidad a través de su calidad inacabada: Piepmeier (2009) explica cómo el "desorden desechable" de los fanzines inicia conversaciones entre el creador y el lector. En cambio, un dibujo es una imagen estática cuyo valor depende de su carácter fijo (Berger, 1976). La finalidad de mi obra de arte puede haber disuadido a los participantes de sugerir cambios. Sin embargo, cuando los dibujos se cortaban para las actividades del taller, su cualidad de acabados se disolvía y los participantes compartían libremente sus historias de volcanes a través de ellos. Esta diferencia en la participación invitada por la creación de fanzines y la ilustración refleja las tensiones entre mis papeles como investigadora y como artista, pero también ofrece diferentes formas de abordar mis preguntas de investigación. El objetivo de mi investigación era amplificar las voces de las personas marginadas, y los métodos participativos dirigidos por la comunidad ofrecen los mejores resultados para comprender los perfiles de riesgo de desastres de las comunidades (van Niekerk y Annandale, 2013). Sin embargo, los artistas suscitan el diálogo compartiendo su visión, haciendo que su papel sea fundamental: "siempre estamos observando la relación entre las cosas y nosotros mismos" (Berger, 1972). Mi doble papel suscitó preguntas: ¿los participantes eran colaboradores o meros críticos? Combinar la creación de fanzines y la ilustración dificultaba la definición. Cada método prometía formas de abordar mis preguntas de investigación: los talleres participativos proporcionaban materiales y espacios para que la gente compartiera creativamente historias de desastres con los demás, mientras que, en las consultas, las ilustraciones provocaban un diálogo más profundo con los individuos sobre sus recuerdos. En el futuro, la combinación de estos métodos para comunicar experiencias de desastres podría abordar la ambigüedad de la implicación de los participantes invitando a una mayor colaboración desde el principio (p. ej., consultas iniciales en grupo sobre el contenido de los fanzines) o limitando el papel del investigador (p. ej., separando los papeles de investigadora y artista). Sin embargo, el deseo de trabajar de forma creativa motiva a muchos investigadores a utilizar estos métodos (Sou et al., 2021). Aunque los investigadores que deseen utilizar prácticas creativas deben adecuar sus habilidades al contexto de su investigación, no solo al entusiasmo (Miller & Brockie, 2015), las respuestas de los participantes en las primeras consultas (pág. 11) sugieren que era apropiado explorar mi habilidad artística en Fuego.

El fanzine y la ilustración ofrecen diferentes medios de representación y niveles de participación. La representación abarca tanto el retrato como el ser retratado. El dibujo es representación, un intento de descubrir o describir la perspectiva del artista, cómo se ven las cosas (Berger, 1972). Aunque los fanzines pueden implicar representación en ambos sentidos, su valor reside a menudo en ofrecer a las comunidades marginadas medios para retratarse a sí mismas en sus propios términos (Gardner, 2023; este estudio, págs. 6 – 7). La dificultad de responder a la pregunta "¿De quién es la visión representada en el fanzine?" revela tensiones entre mis elecciones artísticas y las aportaciones de la comunidad. El comentario "Esta historia no es de nosotros" (pág. 18) indica que esta mujer no sentía conexión con el folleto y las historias que representaba y sugiere que la representación por sí sola es insuficiente para comunicar el riesgo volcánico a las poblaciones de riesgo. Esta interacción reveló una limitación clave: al no haber narradores de fanzines presentes, no hubo diálogo directo entre los supervivientes del desastre y las poblaciones en riesgo. La investigación muestra que la comunicación eficaz del riesgo a las poblaciones vulnerables requiere una participación local continua (Mercer et al., 2008), por lo que estas limitaciones podrían abordarse garantizando la presencia de narradores de fanzines en tales interacciones. Las personas mayores de Fuego deseaban compartir sus historias con las generaciones más jóvenes para informarles sobre los riesgos de Fuego y, en la medida de lo posible, adoptar medidas preventivas (véase **SM-2**, págs. 26 – 27). Una amplia investigación explora los "objetos-límite": resultados de métodos participativos basados en las artes que tienden puentes entre mundos sociales e inspiran la acción:

"Los objetos-límite suelen ser obras de arte materiales, realizadas en colaboración por participantes que viven en situaciones marginales, cuyos rostros y voces no se ven y son desconocidos. Los objetos suelen exponerse en espacios o sesiones públicas para intercambiar conocimientos con las partes interesadas y crear empatía sobre el conocimiento experiencial."

Groot & Abma (2021), pág. 2

Los objetos límite permiten una conexión genuina, pero requieren que los investigadores creen espacios para un diálogo equitativo (Groot & Abma, 2021). El taller de Siquinalá, en el que los narradores utilizaron ilustraciones para compartir sus historias (págs. 16 – 17), ejemplifica un espacio de este tipo. Cuando compartí el fanzine con otra comunidad cercana a Fuego en marzo de 2023, recibió una respuesta entusiasta, con los residentes solicitando un proyecto similar para documentar sus propias experiencias volcánicas. Su deseo de compartir historias con las generaciones más jóvenes se hizo eco de las motivaciones originales de este proyecto, lo que indica el potencial de los fanzines y las ilustraciones como objetos fronterizos para comunicar relatos de catástrofes. Esta labor exige un cuidado ético. La interacción anterior pone de relieve los escollos éticos de la práctica, en particular la ausencia de diálogo y la desigual dinámica de poder en la representación. Aunque incómoda, la interacción iluminó elementos esenciales para la aplicación: facilitar espacios para el diálogo abierto y garantizar la presencia de narradores.

La participación de la comunidad varió a lo largo del proceso de investigación debido en parte a las restricciones de tiempo: tras el primer periodo de trabajo de campo, mis visitas a Fuego fueron breves y mi tiempo compartido con otros proyectos. Esto dificultó el contacto con los participantes, la organización de reuniones y la creación de un sentido de continuidad a lo largo de la duración del estudio. Los facilitadores locales ayudaron inmensamente (pág. 14), pero no siempre estaban disponibles debido a las dificultades para viajar y a preocupaciones familiares. Aunque estos retos logísticos ordinarios son difíciles de evitar en la Guatemala rural, inevitablemente significan que me permití un gran poder sobre el proceso de investigación. Los elementos necesarios para la investigación participativa dirigida por la comunidad – relaciones de confianza a largo plazo, un proceso evolutivo, la inclusión de muchas partes interesadas – también son difíciles de aplicar en el panorama actual de la financiación. Las subvenciones suelen concederse a corto plazo y basarse en supuestos impactos, mientras que: "la naturaleza de la investigación basada en las artes no permite conocer de antemano todos los detalles del proceso, las conclusiones o el posible impacto" (Coemans & Hannes, 2017, p. 42). La beca del consejo de artes que apoya este estudio permitió que el proceso y los resultados del proyecto evolucionaran *in vivo*, e invitó a los becarios a solicitar financiación de seguimiento para sus proyectos: elementos que apoyan la autoevaluación y el estudio longitudinal. Los financiadores que deseen apoyar la investigación participativa podrían considerar cómo las condiciones de la subvención pueden apoyar o inhibir las prácticas necesarias para alcanzar los objetivos de la investigación**.**

Los desafíos que encontré en la participación y representación de la comunidad se relacionan con una lucha clave en la investigación participativa basada en las artes: la "noción de empoderamiento" Coemans & Hannes (2017), o la dificultad de traducir los ideales de empoderamiento de la comunidad en la práctica. En primer lugar, tuve que reconocer las limitaciones del estudio: cualquier empoderamiento que las personas obtuvieran estaría restringido dentro de las inseguridades políticas, sociales y económicas más amplias de Guatemala: "un proyecto por sí solo [no puede] transformar radicalmente ni al individuo ni al sistema dentro del cual tienen que operar" (McKean, 2006, p. 320, citado en Coeman & Hannes (2017), p. 41). Si bien la representación de las experiencias de desastre es vital, el éxito de la reducción del riesgo en Fuego requiere la integración institucional local, uno de los aspectos más difíciles de la implementación (Few et al., 2022). Aunque era consciente de las relaciones de poder locales (esenciales para una investigación participativa eficaz de la RRD (Mercer et al., 2008)), fue muy difícil incluir a los participantes en todas las fases y dar voz a todas las personas con las que trabajé, no sólo a las más elocuentes o seguras de sí mismas. El control que ejercía sobre la investigación y los procesos creativos me convertía a menudo en la autoridad que tomaba las decisiones, lo que planteaba problemas éticos. Algunas decisiones destinadas a maximizar la representación reforzaron involuntariamente esa autoridad. Por ejemplo, aunque el fanzine incluía los recuerdos de los participantes citados textualmente – “El objetivo era presentar las voces de los participantes lo más crudas posible (Valli, 2021) – yo decidía en última instancia qué recuerdos incluir en el fanzine (pág. 13). Aunque la investigación participativa implica inherentemente cierto desequilibrio de poder (Few et al., 2022), hice varios esfuerzos para diversificar la participación y mitigar este desequilibrio. En el taller de Panimaché Dos y en varias consultas, contraté a un miembro de la comunidad como facilitador. Aunque siete años de trabajo en Fuego me han proporcionado un rico conocimiento de la cultura y la lengua local, sigo siendo una forastera. Los facilitadores enriquecieron las conversaciones aportando conocimientos sobre el contexto local, lo que contribuyó a crear una atmósfera de confianza y a mitigar los problemas de las relaciones de poder entre investigadores y participantes (Mercer et al., 2008; Le De et al., 2014). Los talleres se diseñaron para que las personas utilizaran sus voces para dirigir la actividad y contar sus propias historias. Mientras tanto, intenté incluir las voces de las personas en las consultas a través de su narración de los primeros bocetos y las ilustraciones posteriores, y se solicitaron sus opiniones para comentar y revisar estas ilustraciones. Entre los dibujos que se modificaron en respuesta a las opiniones recibidas figuran las páginas 8 y 29, y entre los que se añadieron, las páginas 10 y 13. Los métodos participativos basados en las artes pueden facilitar el empoderamiento de la comunidad mediante la apropiación del proceso de investigación (Valli, 2021) o la representación de experiencias individuales de desastres (Miller & Brockie, 2015). Si bien los comentarios sugieren que los métodos presentados aquí requieren perfeccionamiento (págs. 22 – 25), en las respuestas de algunos participantes (págs. 11, 18, 35) aparecen pruebas de empoderamiento a través de la representación. Estos métodos combinados son prometedores para responder a las dos preguntas de la investigación. Las iteraciones futuras podrían incorporar sesiones de intercambio de conocimientos dirigidas por los participantes, utilizando los fanzines como objetos-límite para compartir la narrativa de la comunidad. La experiencia indica que la comunidad está interesada en este tipo de foros de comunicación de riesgos entre iguales (nota a pie de pág. 9).

Además de los retos que plantea la participación y la representación de la comunidad, trabajar como artista e investigador ofrece ventajas únicas. Mientras que los investigadores han utilizado diversos métodos basados en las artes para comprender las múltiples experiencias de los desastres (**Métodos basados en las artes para explorar la respuesta y la recuperación a los desastres,** pág. 6), la ilustración y la creación de fanzines siguen estando poco exploradas a pesar de sus probadas ventajas en la representación (Berger, 1972; Gardner, 2023). Tanto la habilidad investigadora como la creativa requieren reflexión, práctica y conocimiento contextual. A través de mis años de trabajo con la gente en Fuego y haciendo arte, yo era el único capaz de llevar mis habilidades tanto en la investigación y el arte a un contexto en el que estaba bien informado, sensible al trauma, y capaz de amplificar las voces de la gente de desastre. Aunque no conozco los criterios existentes para evaluar la calidad y el impacto de las ilustraciones para explorar la experiencia del desastre, las indicaciones para un enfoque poético proporcionan una posible guía de evaluación:

Desde un punto de vista crítico, a la hora de evaluar la calidad y el impacto de un enfoque poético, Sparkes y Douglas (2007) sugieren cuatro pautas a tener en cuenta: el mérito estético (p. ej., tienen forma artística, crean conexiones evocadoras), su impacto (p. ej., afectan al lector emocional e intelectualmente, generan nuevas preguntas, mueven a la gente a la acción), autenticidad ontológica y educativa (por ejemplo, estimulan la reflexión de los participantes en la investigación y de otros) y, por último, una consideración de las cuestiones éticas (por ejemplo, participantes comprometidos en el proceso).

Miller & Brockie (2015), pág. 106

En una entrevista, un participante recordó haber admirado las fuentes incandescentes de Fuego en los años anteriores a sus destructivas erupciones de los años sesenta y setenta. Desarrollé su testimonio en una ilustración detallada que incluía su descripción textualmente **(Figura 7)**. Cuando le regalé esta obra de arte en una consulta posterior, expresó una mezcla de alegría y tristeza, describió cómo la lámpara de parafina le evocaba recuerdos del uso de velas en la oscuridad y expresó su esperanza de que los dibujos pudieran compartirse con las generaciones más jóvenes para informarles de la historia de Fuego. Esta y otras consultas (págs. 11, 18, 35) abordan las cuatro consideraciones para la investigación poética y dan apoyo cualitativo a los métodos visuales para fomentar la representación y un diálogo más profundo del desastre (Tatham-Fashanu (2023)). Las futuras iteraciones de estos métodos podrían incluir o adaptar el enfoque poético mencionado anteriormente para evaluar su calidad e impacto de forma más exhaustiva, incluido su significado emocional.


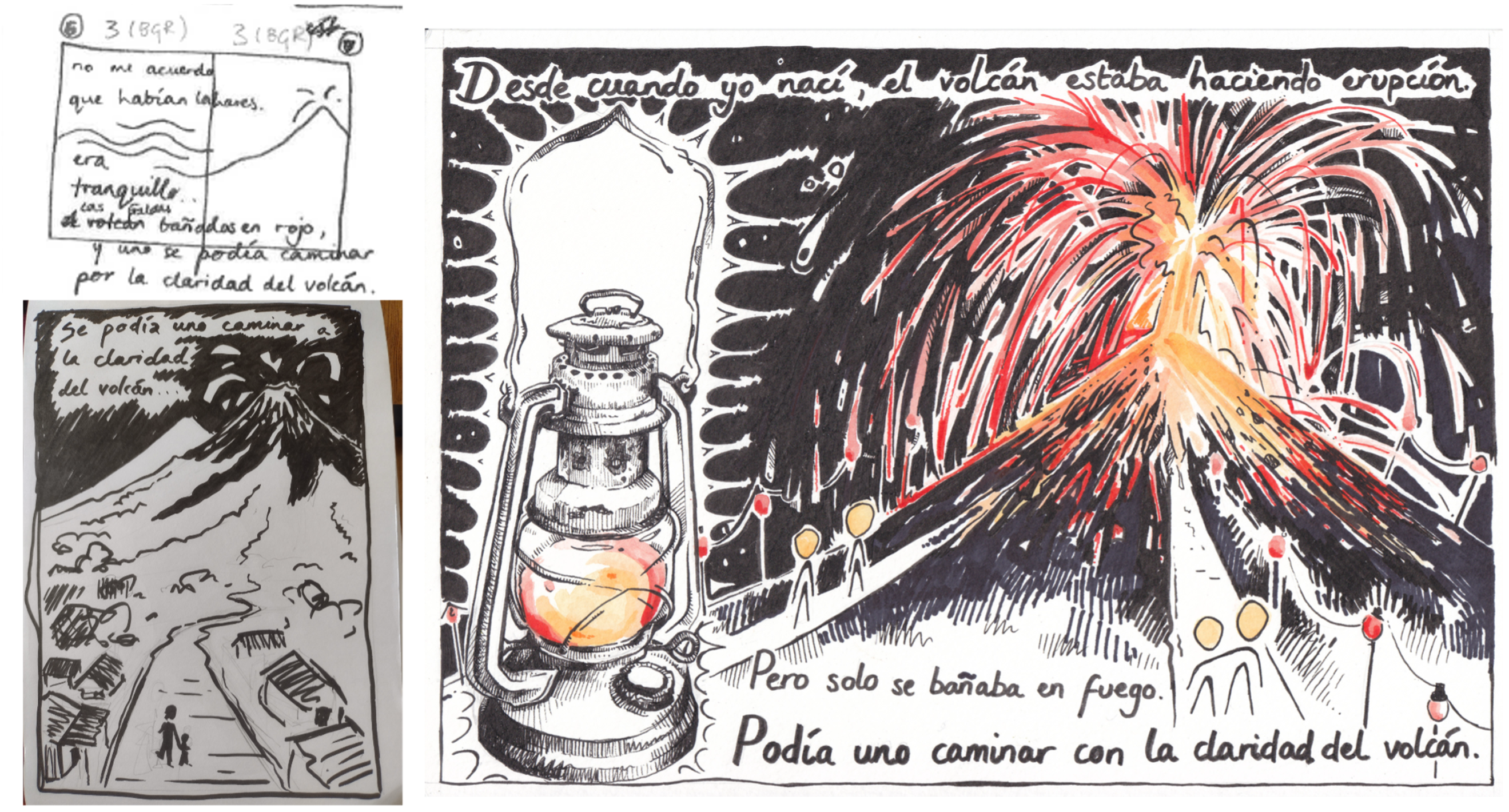


**Figura 7:** desarrollo de una de las páginas del fanzine, desde (arriba a la izquierda) un primer garabato en storyboard hasta (abajo a la izquierda) un boceto más evolucionado y (derecha) la versión final en tinta y acuarela.

Coeman y Hannes (2017) instan a los investigadores que utilizan métodos basados en las artes a reflexionar tanto sobre el proceso como sobre el resultado. Mi doble perspectiva de investigadora-artista me permitió matizar ambos aspectos. En este estudio, la relación entre los niveles de participación y el empoderamiento demostró ser no lineal: las actividades muy participativas no garantizaban el compromiso (pág. 17), mientras que los enfoques menos participativos a veces lograban una representación significativa. Esto sugiere que, para los investigadores que deseen utilizar métodos basados en las artes con las comunidades, el objetivo no es simplemente "llegar al peldaño más alto de la escalera participativa" **(Figura 6)**, sino evaluar los diferentes beneficios que ofrecen los enfoques más y menos participativos. Mientras que las consultas produjeron el fanzine, los talleres crearon un rico espacio para contar historias dando prioridad al proceso sobre los resultados. Los talleres también produjeron artefactos no convencionales creados conjuntamente (véase **SM-3**). Esto coincide con el argumento de Valli (2021) de que la combinación de diferentes niveles de participación en el diseño de la investigación puede optimizar los resultados de la misma.

Aunque los métodos participativos basados en las artes ofrecen nuevas formas de procesar los recuerdos traumáticos (Huss et al., 2016), hay que tener cuidado para evitar volver a traumatizar a los participantes. Aunque los participantes encontraron significado en la narración visual, y las ilustraciones profundizaron nuestro diálogo, la representación del trauma planteó preocupaciones éticas de causar daño al reanimar sus recuerdos dolorosos. No estaba segura de si el hecho de que una persona ajena ilustrara esos recuerdos sería beneficioso (al registrar la experiencia vivida por las personas) o perjudicial (al evocar el dolor). Al recibir las ilustraciones de sus recuerdos, los participantes a menudo expresaban simultáneamente dolor y agradecimiento. Mi doble papel de artista-investigadora, junto con mi larga relación con la gente de Fuego, me permitió manejar con sensibilidad estas conversaciones íntimas. Las primeras consultas (pág. 11) sugirieron que la representación visual sería valiosa, mientras que mi experiencia en investigación ayudó a crear espacios seguros para estas conversaciones.

"La atención es el principio de la devoción" escribió la poeta Mary Oliver (Oliver, 2019, p. 5); ella sitúa la atención como el trabajo esencial de la vida. Prestar atención dos veces fue una ventaja única para mi doble papel de artista-investigadora. Al escuchar las historias que la gente contaba sobre Fuego, aprendí cómo habían experimentado sus erupciones e impactos. Documenté estas experiencias para amplificar sus voces, comprender cómo su experiencia pasada influye en la respuesta actual a la crisis y preservar los recuerdos que se desvanecen (la mayoría de los testigos de estas erupciones tienen entre 60 y 70 años). El dibujo complementó este trabajo de investigación como otro medio de comprender y preservar experiencias al borde de la memoria viva. Pero el dibujo ofrecía algo más. Los habitantes de la zona compartieron sus recuerdos del Fuego con un lenguaje vívido, empleando gestos y efectos sonoros al describir los momentos culminantes. En nuestro diálogo se produjo una especie de magia: un participante que narraba su experiencia podía evocar una poderosa imagen dentro de mi cabeza que yo podía trasladar al papel. Mis ilustraciones intentaban transmitir esa "visión que precede a las palabras y que nunca puede ser cubierta por ellas" (Berger, 1972, p. 1). Cada ilustración iba acompañada de una cita literal para rendir homenaje a la interacción que la produjo. Aunque este enfoque requería una menor implicación por parte de los participantes, mostraba el enorme poder de la ilustración para representar la experiencia del desastre: mirar como un acto de elección, dibujar para honrar la conexión entre el investigador y el participante, y dibujar para desafiar la desaparición. Un dibujo "...implica, deriva y remite a muchas experiencias previas de mirar. En el instante de ver un árbol se establece una experiencia vital. Así es como el acto de dibujar rechaza el proceso de las desapariciones y propone la simultaneidad de una multitud de momentos" (Berger, 1976, p. 43 – 44). La ilustración para desafiar la desaparición se adaptaba a un proyecto basado en la experiencia vivida del desastre entre las personas mayores, mientras que la ilustración desde la mirada como acto de elección comunicaba a la población local mi continuo interés por trabajar con ellos y registrar sus experiencias. La ilustración para preservar el pasado y abrir conversaciones sobre las historias de vida de los participantes es paralela a otras investigaciones que utilizan métodos creativos para hablar con sensibilidad de recuerdos traumáticos (Marsh et al., 2020). Del mismo modo, un "fanzine posiciona [a los lectores] como amigos, iguales, miembros de una comunidad encarnada que forman parte de una conversación con el creador del fanzine" (Piepmeier, 2009, p. 71). Mi doble papel de investigadora-artista ofrecía múltiples medios para conectar con los participantes y demostrar mi compromiso de registrar sus historias. Para los investigadores que han dedicado tiempo a trabajar con comunidades de riesgo, la creación de fanzines y la ilustración ofrecen poderosas formas de "prestar atención" de forma creativa para honrar esa conexión.

Sou et al. (2021) abogan por la ilustración gráfica de la investigación para fomentar la influencia de los participantes y crear productos de investigación accesibles. Por estas razones, y las expresadas anteriormente, emprendí este difícil y gratificante proyecto. Este proyecto se benefició de la experiencia de muchos colegas en Ixchel, y profundizó mis relaciones existentes con la gente alrededor de Fuego. Guiada por el llamamiento de Coemans & Hannes (2017) a explorar tanto el proceso como los resultados, comparto estas ideas para otros investigadores de desastres que estén considerando el uso de métodos participativos creativos:

1. **Considera el control.** Tuve mucho control en la investigación y los procesos creativos de la creación del fanzine. En las actividades de taller, los participantes se implicaron mucho y dirigieron el proceso; también tuve que aceptar ceder el control sobre los resultados de la investigación. Los distintos niveles de participación de la comunidad ofrecen diferentes ventajas. ¿Cuánto poder tiene usted (como investigador) en el diseño de su estudio? ¿En qué medida invitará a los participantes a decidir la dirección de la investigación y qué ganaría el proceso si usted cediera algo de poder?
2. **¿Proceso o resultado?** La elaboración del fanzine se centró en la creación de un producto tangible. Las actividades participativas se centraron en el proceso de crear comprensión a través de la narración de historias en un entorno de confianza. Otros estudiosos señalan las diferentes virtudes del diseño de la investigación centrado en el proceso y en el resultado. ¿Qué partes del diseño de su estudio se dedican al proceso y cuáles a los resultados? ¿Cambia la forma de representación del proceso al resultado?
3. **Considere la inversión y el valor de la lentitud académica.** Este artículo resume un proyecto de tres años que incluye copiosas notas, docenas de conversaciones y múltiples bocetos, ninguno de los cuales aparecerá nunca como resultado de la investigación. El proyecto me pareció lento, largo e intensamente gratificante; redobló mi conexión con Fuego y sus gentes. Este proyecto fue posible gracias al alcance de Ixchel y a la flexibilidad que ofrecía la beca del consejo de artes. ¿Puedes invertir en un proyecto participativo? ¿Cuáles vías de financiación pueden apoyar su proyecto?
4. **¿Cuántas voces?** A pesar de mi experiencia de trabajo en Fuego, luché por dar voz a todo el mundo, desde invitar a hablar a los tímidos hasta gestionar "protagonistas" que siempre hablaban, a veces por encima de los demás. Es importante ampliar activamente la participación, aceptando al mismo tiempo que el compromiso de la comunidad se consigue a menudo a través de unos pocos individuos motivados. Contraté a un facilitador local y trabajé con los grupos comunitarios existentes para abordar estas cuestiones. ¿Qué métodos empleará usted para maximizar la representación de muchas voces? ¿Cómo puede trabajar usted con individuos motivados para ampliar la participación?
5. **Tener en cuenta las diferencias culturales.** Las ideas de este proyecto me resultan familiares gracias a mi red de investigación y a la cultura británica (en concreto, la cultura local de Bristol). En cambio, a veces me confundían cuando las comentaba con la gente de Fuego. Por lo tanto, fue necesario un esfuerzo para traducir las ideas a un lenguaje más familiar para la gente de Fuego. Dedicar algo de tiempo a la traducción de ideas culturalmente desconocidas es esencial.
6. **Todo el mundo necesita sentirse importante.** Algunos momentos fuertes de este proyecto se produjeron en las consultas individuales, cuando regalé a una persona una ilustración de su recuerdo personal. Fueron momentos de gran emoción para mí y, a menudo, para el participante. Estos encuentros coinciden con las reflexiones de otros investigadores sobre la importancia de reconocer las historias individuales de desastres de las personas. Considere cómo el diseño de su investigación hace que los participantes se sientan importantes.
7. **La importancia de volver.** Independientemente de sus respuestas al proceso de investigación, la gente de Fuego estaba encantada cuando volví. Siempre que sea posible, es importante volver: invertir tiempo en estar juntos evita algunas de las peores tendencias de la investigación explotadora. Volver también honra la relación que se establece con los participantes al demostrar que sus historias y su compañía también tienen significado para ti.

Este debate tenía como objetivo evaluar críticamente mi proceso de investigación y mi éxito a la hora de abordar mis objetivos de investigación. El ejercicio reveló cómo reproduje algunos retos conocidos de la investigación participativa, especialmente a través del control que ejercía como investigadora y artista. El ejercicio también sugirió que, aunque necesitados de refinamiento, la ilustración y la creación de fanzines tienen valor como métodos de representación para compartir historias de desastres. La pregunta "¿De quién es la representación?" es muy pertinente en los estudios en los que los investigadores trabajan de forma creativa. Abordar esta cuestión requiere que los investigadores aclaren sus ideas sobre cómo se logrará la capacitación de los participantes y permite reflexionar posteriormente sobre si esto se llevó a cabo en la práctica. Mi doble papel de investigador-artista complicó la implicación y representación de los participantes, pero también ofreció una rica conexión con la gente de Fuego y la posibilidad de alcanzar nuestro objetivo compartido de amplificar las voces marginadas y comunicar el desastre a las personas en situación de riesgo, como se refleja en esta entrevista a un participante en marzo de 2024:

Participante (Morelia): Agradecido. Muy, muy, profundamente por este material que usted nos trajo. Pero esperemos en Dios que nosotros muramos en paz … y pedirle a Dios por la generación siguiente. Para eso sirve ese material, para eso sirven ustedes que dan esa plática, a los que vendrán y los jóvenes que están estudiando. Que se capaciten con relación a esto, lo que hemos vivido nosotros, porque esta generación ya va de salida, ya nosotros ya somos historia. Próximamente vamos a hacer historia. Entonces es necesario dejarle la plática o el conocimiento a los demás. Uno de estos se los voy a dejar a mis hijos. Vamos a compartirle a mis hijos para que ellos lean y … es una historia plasmada en un libro que precisamente de esta área. Qué bueno, seño. Y yo a usted le agradezco mucho por este material pues. Nos va a ayudar un montón, si no a nosotros, a los demás que vendrán. Y eso es maravilloso.

**Conclusiones**

Este artículo describe el proceso de creación de un fanzine sobre las experiencias de la población local ante las erupciones del Volcán de Fuego (Guatemala) en la segunda mitad del siglo XX. Se exploraron la ilustración y la creación de fanzines como herramientas para registrar las experiencias de la gente sobre el desastre volcánico y compartirlas con las personas en riesgo de su comunidad. La investigación incluyó una serie de consultas y talleres participativos entre 2021 y 2024. Los problemas de implicación y representación de los participantes sugieren la necesidad de perfeccionar la metodología. Sin embargo, la ilustración mostró potencial para explorar con sensibilidad los recuerdos del desastre, mientras que las actividades participativas permitieron a la gente compartir de forma creativa historias de Fuego entre comunidades y generaciones. Una autoevaluación crítica revela cómo mi doble papel de investigadora-artista contenía tensiones que complicaban mis objetivos de investigación, pero también ofrecía ventajas únicas para trabajar de forma sensible con el trauma, preservar los recuerdos que envejecen y enriquecer las relaciones existentes "prestando atención dos veces". Estas reflexiones aportan ideas a otros investigadores que consideren los métodos basados en las artes como medios para representar y amplificar las voces de las personas afectadas por desastres.

**Declaraciones**

**Aprobación ética y consentimiento para participar**

Este estudio se sometió a revisión ética y fue aprobado por el Comité de Ética de la Investigación de la Universidad de Bristol (ID de proyecto 246). Se solicitó y obtuvo el consentimiento para participar de todos los participantes.

**Autorización por publicación**

No aplicable.

**Disponibilidad de datos y materiales**

El fanzine y los materiales de apoyo a este trabajo están disponibles en la **Información suplementaria.** Por razones éticas, los datos de las entrevistas no están a disposición del público.

**Intereses contrapuestos**

El autor principal no tiene intereses contrapuestos que declarar.

**Financiación**

A.K.N. desea agradecer el apoyo de un fondo semilla de la Cuenta Aceleradora de Impacto AHRC de la Universidad de Bristol (ciclo 2022 – 2025), *Using creative methods to co-develop experiences of eruption at Volcán de Fuego, Guatemala* (A100416-110). A.K.N. también desea agradecer el apoyo del proyecto de investigación interdisciplinario del GCRF, *Ixchel: Building understanding of the physical, cultural and socio-economic drivers of risk for strengthening resilience in the Guatemalan cordillera* (NE/T010517/1).

**Contribuciones de los autores**

A.K.N. hizo todos los bocetos, dibujos e ilustraciones, realizó y codificó las entrevistas, organizó las consultas y los talleres, contrató a los animadores de los talleres y redactó el manuscrito.

**Agradecimientos**

Este estudio se concibió en conversación con otros investigadores de *Ixchel*. Como participante en un gran grupo de trabajo interdisciplinario centrado en la RRD en Guatemala, reconozco que este amplio contexto de trabajo enriquece los resultados individuales, y que las publicaciones resultantes se benefician del contexto proporcionado en un espíritu de intercambio abierto y generoso entre los miembros del grupo. Me gustaría dar las gracias en particular a Teresa Armijos Burneo, Cristina Sala Valdez y Lina Rabe por compartir las ideas de su amplia experiencia colectiva. Su sensibilidad y cuidado en su trabajo con las mujeres de Fuego es profundamente inspirador, y agradezco su invitación a unirme a su proceso en Siquínala. Gracias a la cuenta IAA del AHRC por el apoyo financiero para explorar una idea de investigación inusual y desarrollarla *in vivo*. Me gustaría dar las gracias especialmente a Eliza Calder y Lisa Mackenzie, cuyos comentarios iluminaron el valor de ser tanto artista como investigadora. Ashrika Sharma, Aracely Martínez, Ale Colom y Janina Engler compartieron reflexiones sobre el poder y la participación que sirvieron de base para la discusión de este artículo. Muchas gracias a los revisores y al editor, cuyos atentos comentarios mejoraron sustancialmente este manuscrito. Por último, estoy profundamente agradecida a la gente de Fuego por su generosidad al compartir sus historias. El fanzine Fuego también representa mi gratitud hacia ellos por su tiempo y amistad a lo largo de siete años.

**Información complementaria**

1. **SM-1:** Guión gráfico del Zine
2. **SM-2:** Segunda edición del fanzine Fuego
3. **SM-3:** Obras creadas en talleres participativos (**SM-3a**) y en consulto individual (**SM-3b**)
4. **SM-4:** Versión en español de este manuscrito

**Referencias**

Arnstein S R (1969) A ladder of citizen participation. Journal of the American Institute of planners 35(4): 216-224.

Berger J (1972) Ways of Seeing. <https://www.ways-of-seeing.com/ch1>. Accessed 9 Nov 2024

Berger J (1976) Drawn To That Moment. In: Berger On Drawing (2005). <https://www.spokesmanbooks.com/Spokesman/PDF/90Berger.pdf> . Accessed 9 Nov 2024

Bristol Doctoral College (BDC) blog (2020) Ailsa’s ‘ExPhDition’ — why a PGR illustrated her route to a research degree. <https://bdc.bris.ac.uk/2020/07/30/ailsas-exphdition-why-a-pgr-illustrated-her-route-to-a-research-degree/> Accessed 9 Nov 2024

Bergold J, Thomas S (2012) Participatory research methods: A methodological approach in motion. Historical Social Research/Historische Sozialforschung,191-222.

Childs G, Craig S, Dhakal D N, Donohue M, & Hildebrandt K (2017). Narrating disaster through participatory research: Perspectives from post-earthquake Nepal. Collaborative Anthropologies 10(1): 207-236. <https://doi.org/10.1353/cla.2017.0009>

Coemans S, Hannes K (2017) Researchers under the spell of the arts: Two decades of using arts-based methods in community-based inquiry with vulnerable populations. Educational Research Review, 22:34-49. <https://doi.org/10.1016/j.edurev.2017.08.003>

Coppa F (2006) A brief history of media fandom. In: Hellekson K, Busse K (eds) Fan fiction and fan communities in the age of the Internet. McFarland, North Carolina. <https://books.google.com/books?hl=en&lr=&id=11ODBAAAQBAJ&oi=fnd&pg=PA41&dq=zine+history&ots=3BQeIAaRJ6&sig=6gJQd2PtVyHOWVHRvrbhvZYEyIY&redir_esc=y#v=onepage&q=zine%20history&f=false> . Accessed 29 May 2024.

Cornwall A, Jewkes R (1995) What is participatory research? Social Science & Medicine 41(12):1667-1676. <https://doi.org/10.1016/0277-9536(95)00127-S>

Deterding N M, Waters M C (2021) Flexible coding of in-depth interviews: A twenty-first-century approach. Soc methods & research, 50(2):708-739. <https://doi.org/10.1177/0049124118799377>

Duncombe S. (1997) Notes from the Underground: Zines and the Politics of Alternative Culture. Verso. <https://books.google.co.uk/books?hl=en&lr=&id=l7JYiza7N04C&oi=fnd&pg=PA1&dq=%22stop+consuming+that+which+is+made+for+you%22+duncome&ots=kEjaqYkArj&sig=oPaWRqVa_nV3jfctyKV1YY2u0mM&redir_esc=y#v=onepage&q&f=false> . Accessed 29 May 2024.

Espinoza A E, Osorio-Parraguez P, Posada Quiroga E (2019) Preventing mental health risks in volunteers in disaster contexts: The case of the Villarrica Volcano eruption, Chile. Reduction, 34:154-164. <https://doi.org/10.1016/j.ijdrr.2018.11.013>

Few, R., Barclay, J., & Armijos Burneo, T. (2022). Working with communities on disaster risk research: reflections from cross-disciplinary practice. International Journal of Disaster Risk Reduction, 70. <https://ueaeprints.uea.ac.uk/id/eprint/85037/1/Editorial_for_IJDRR_revised_to_format.pdf>

Gardner, D. (2023) Powerful peculiarity: zines as a tool to represent subjectivity in peripheral communities. <http://hdl.handle.net/20.500.12648/10394>

Groot, B., & Abma, T. (2021). Boundary objects: Engaging and bridging needs of people in participatory research by arts-based methods. International Journal of Environmental Research and Public Health, 18(15), 7903. <https://doi.org/10.3390/ijerph18157903>

Huss E, Kaufman R, Avgar A, Shuker E (2016) Arts as a vehicle for community building and post‐disaster development. Disasters 40(2):284-303. <https://doi.org/10.1111/disa.12143>

Le De L, Gaillard J C & Friesen W (2014) Academics doing participatory disaster research: how participatory is it? Env Haz 14(1):1–15. <https://doi.org/10.1080/17477891.2014.957636>

Legendre, I. (2023). Margins in Motion: Towards a Political History of Zine Culture (Doctoral dissertation). <https://qspace.library.queensu.ca/server/api/core/bitstreams/a7be31cb-8959-446e-ad91-3dfbbfdea586/content> . Accessed 22 Oct 2024.

Marsh H, Armijos Burneo M T, Few R (2020) “Telling it in our own way”: Doing music-enhanced interviews with people displaced by violence in Colombia. New Area Studies 1(1):132-264. <https://ueaeprints.uea.ac.uk/id/eprint/77854/> . Accessed 29 May 2024.

McKean A (2006) Playing for time in ‘the dolls’ house’. Issues of community and collaboration in the devising of theatre in a women's prison. Research in drama education 11(3):313-327. <https://doi.org/10.1080/13569780600900685>

Mercer J, Kelman I, Lloyd K, Suchet‐Pearson S (2008) Reflections on use of participatory research for disaster risk reduction. Area 40(2):172-183. <https://doi.org/10.1111/j.1475-4762.2008.00797.x>

Miller E, Brockie L (2015) The disaster flood experience: Older people's poetic voices of resilience. Journal of Aging Studies 34:103-112. <https://doi.org/10.1016/j.jaging.2015.05.003>

Mountz A, Bonds A, Mansfield B, Loyd J, Hyndman J, Walton-Roberts M, ... Curran W (2015) For slow scholarship: A feminist politics of resistance through collective action in the neoliberal university. ACME 14(4):1235-1259. <https://doi.org/10.14288/acme.v14i4.1058>

Naismith A, Armijos Burneo M T, Barrios Escobar E A, Chigna W, Watson I M (2020) Fireside tales: understanding experiences of previous eruptions and factors influencing the decision to evacuate from activity of Volcán de Fuego. Volcanica 3(2):205–226. <https://doi.org/10.30909/vol.03.02.205226>

Naismith A, Escobar Wolf R, Bartel B, et al (2024) West Side Story: Evaluating impacts of 20th century eruptions of Volcán de Fuego (Guatemala) on local lives and livelihoods. Manuscript in preparation.

Nalla V, Ranjit N, Udupa Y, Madhayan M, Arvind J, Jain G, Malladi T (2022) Afterwards – Graphic Narratives of Disaster Risk and Recovery from India. Indian Institute for Human Settlements. <https://doi.org/10.24943/9788195648559>

Nkombi Z, Wentink GJ (2022) The role of public participation in disaster risk reduction initiatives: The case of Katlehong township. Jamba 28 14(1):1203. doi: 10.4102/jamba.v14i1.1203

Oliver, M. (2019). Upstream: selected essays. Penguin.

Paton D, Buergelt P, Pavavalung E, Clark K, Jang L J, Kuo G (2022) All Singing from the Same Song Sheet: DRR and the Visual and Performing Arts. In: James H, Shaw R, Sharma V, Lukasiewicz A (eds) Disaster Risk Reduction in Asia Pacific. Palgrave Macmillan, Singapore. <https://doi.org/10.1007/978-981-16-4811-3_7>

Piepmeier, A. (2009). Girl Zines: Making Media, Doing Feminism. New York, USA: New York University Press. <https://doi.org/10.18574/nyu/9780814768501.001.0001>

Romano A (2023) Exploring the “Zine”. <https://www.salemart.org/wp-content/uploads/2023/08/Adam-Romano-Exploring-the-Zine-Final-Draft-PDF.pdf> . Accessed 29 May 2024.

Schumann R L, Binder S B, Greer A (2019) Unseen potential: photovoice methods in hazard and disaster science. GeoJournal 84:273–289. <https://doi.org/10.1007/s10708-017-9825-4>

Sparkes, A.C., & Douglas, K. (2007). Making the case for poetic representations:

An example in action. The Sport Psychologist, 21, 170–190.

Sou G (2023) Wiley Lecture 2022. Communicating climate change with comics: Life beyond apocalyptic imaginaries. Geographical Research 61(3):320–332. <https://doi.org/10.1111/1745-5871.12592>

Sou G, Douglas J C, Díaz-Basteris F (2021) After Maria by Gemma Sou and John Cei Douglas. Studies in Comics 12:129-135. <https://doi.org/10.1386/stic_00055_3>

Sou G, Hall S M (2021) Communicating crisis research with comics: representation, process, and pedagogy. In: Kara H, Khoo SM (eds) Qualitative and Digital Research in Times of Crisis: Methods, Reflexivity, and Ethics. Policy Press. <https://doi.org/10.51952/9781447363828.ch006>

Sou G, Hall S M (2023) Comics and Zines for Creative Research Impact: Ethics, Politics and Praxis in Geographical Research. ACME 22(1):817–841. <https://doi.org/10.7202/1098037ar>

Stone J, Barclay J, Simmons P et al (2014) Risk reduction through community-based monitoring: the vigías of Tungurahua, Ecuador. J Appl. Volcanol. 3(11). <https://doi.org/10.1186/s13617-014-0011-9>

Tatham-Fashanu C (2023) Enhancing participatory research with young children through comic-illustrated ethnographic field notes. Qualitative Research 23(6):1714-1736. https://doi.org/10.1177/14687941221110186

Triggs T (2006) Scissors and glue: Punk fanzines and the creation of a DIY aesthetic. J Design History 19(1):69-83. <https://www.jstor.org/stable/3838674> Accessed 29 May 2024.

Up Our Street (2021, Spring). “Can You Spot These Around Easton?” (pg. 16) <https://elhnm.sharepoint.com/sites/EastsidePublications/Up%20Our%20Street%20magazine/Forms/AllItems.aspx?id=%2Fsites%2FEastsidePublications%2FUp%20Our%20Street%20magazine%2FSpring%202021%2Epdf&parent=%2Fsites%2FEastsidePublications%2FUp%20Our%20Street%20magazine&p=true&ga=1> Accessed 15 Jun 2024.

Valli C (2021) Participatory dissemination: bridging in-depth interviews, participation, and creative visual methods through Interview-Based Zine-Making (IBZM). Fennia 199(1):25–45. <https://doi.org/10.11143/fennia.99197>

Van Niekerk D, & Annandale E (2013) Utilising Participatory Research Techniques for Community-Based Disaster Risk Assessment. Intl Journ of Mass Emergencies & Disasters 31(2):160-177. <https://doi.org/10.1177/028072701303100203>

Wisner B, Gaillard J C, Kelman I (2012). Handbook of hazards and disaster risk reduction. Routledge.

1. Mi primer fanzine sobre volcanes describía la erupción del Volcán Santa María en 1902 y la formación del Volcán Santiaguito a través de la leyenda de Juan Noj. El fanzine pretendía comunicar conocimientos vulcanológicos de forma creativa y se compartió en un simposio conmemorativo del centenario de la erupción del Volcán Santiaguito en 1922. [↑](#footnote-ref-1)
2. Por ejemplo, concerté una consulta con un participante en Panimaché Dos. Esperaba una conversación cara a cara, pero llegué a la reunión y me encontré con más de 30 personas, entre ellas el grupo local de voluntarios para la reducción de desastres ("COLRED"). Mi contacto había invitado a otros miembros de la comunidad a la reunión para que él y sus compañeros pudieran compartir sus experiencias sobre las erupciones de Fuego en los años sesenta y setenta. [↑](#footnote-ref-2)
3. Morelia era antiguamente una *finca* privada que producía café, una historia que este participante conocía y me contó. Incluí granos de café en el dibujo para reconocer esta historia. [↑](#footnote-ref-3)
4. Sou desarrolló su cómic con familias de bajos ingresos en un barrio de Puerto Rico afectado por el huracán María en 2017, mientras que Tatham-Fashanu trabajó con un grupo diverso de niños de entre 4 y 6 años en una escuela primaria del norte de Inglaterra. [↑](#footnote-ref-4)
